# Exosomal HMGA2 protein from EBV-positive NPC cells destroys vascular endothelial barriers and induces endothelial-to-mesenchymal transition to promote metastasis

**DOI:** 10.1038/s41417-022-00453-6

**Published:** 2022-04-06

**Authors:** Deng-Ke Li, Xing-Rui Chen, Li-Na Wang, Jia-Hong Wang, Ji-Ke Li, Zi-Ying Zhou, Xin Li, Lin-Bo Cai, Shui-Sheng Zhong, Jing-Jing Zhang, Yu-Mei Zeng, Qian-Bing Zhang, Xiao-Yan Fu, Xiao-Ming Lyu, Min-Ying Li, Zhong-Xi Huang, Kai-Tai Yao

**Affiliations:** 1 Guangdong Provincial Key Laboratory of Tumor Immunotherapy, Cancer Research Institute, School of Basic Medical Sciences, Southern Medical University, Guangzhou, 510515 China; 2 Guangzhou First People’s Hospital, School of Medicine, Southern China University of Technology, Guangzhou, 510180 China; 3 Shenzhen Key Laboratory of Viral Oncology, the Clinical Innovation & Research Center (CIRC), Shenzhen Hospital, Southern Medical University, Shenzhen, 518110 China; 4 Guangdong Sanjiu Brain Hospital, Guangzhou, 510510 China; 5 Department of Radiotherapy, Tumor Hospital of Zhongshan People’s Hospital, Zhongshan, 528403 China; 6 Department of Pathology, Tumor Hospital of Zhongshan People’s Hospital, Zhongshan, 528403 China; 7 Department of Otorhinolaryngology Head and Neck Surgery, General Hospital of Southern Theater Command, People’s Liberation Army of China, Guangzhou, 510010 China; 8 Department of laboratory medicine, The Third Affiliated Hospital, Southern Medical University, Guangzhou, 510000 China

**Keywords:** Cancer microenvironment, Metastasis

## Abstract

Increased vascular permeability facilitates metastasis. Cancer-secreted exosomes are emerging mediators of cancer-host crosstalk. Epstein-Barr virus (EBV), identified as the first human tumor-associated virus, plays a crucial role in metastatic tumors, especially in nasopharyngeal carcinoma (NPC). To date, whether and how exosomes from EBV-infected NPC cells affect vascular permeability remains unclear. Here, we show that exosomes from EBV-positive NPC cells, but not exosomes from EBV-negative NPC cells, destroy endothelial cell tight junction (TJ) proteins, which are natural barriers against metastasis, and promote endothelial-to-mesenchymal transition (EndMT) in endothelial cells. Proteomic analysis revealed that the level of HMGA2 protein was higher in exosomes derived from EBV-positive NPC cells compared with that in exosomes derived from EBV-negative NPC cells. Depletion of HMGA2 in exosomes derived from EBV-positive NPC cells attenuates endothelial cell dysfunction and tumor cell metastasis. In contrast, exosomes from HMGA2 overexpressing EBV-negative NPC cells promoted these processes. Furthermore, we showed that HMGA2 upregulates the expression of Snail, which contributes to TJ proteins reduction and EndMT in endothelial cells. Moreover, the level of HMGA2 in circulating exosomes is significantly higher in NPC patients with metastasis than in those without metastasis and healthy negative controls, and the level of HMGA2 in tumor cells is associated with TJ and EndMT protein expression in endothelial cells. Collectively, our findings suggest exosomal HMGA2 from EBV-positive NPC cells promotes tumor metastasis by targeting multiple endothelial TJ and promoting EndMT, which highlights secreted HMGA2 as a potential therapeutic target and a predictive marker for NPC metastasis.

## Introduction

Cancer metastasis is a complex, multistep process in which tumor cells detach from the primary site, migrate and invade the extracellular matrix, intravasate into vessels, survive in the bloodstream, extravasate from vessels, seed in target organs and grow to form metastatic nodules [1]. The traversal of tumor cells through the vascular barrier is a crucial step in the metastasis process [2, 3]. Cell-to-cell junctions between endothelial cells, which are critical in maintaining vascular integrity, consist of adherens junctions and tight junctions (TJs). The endothelial junction complex comprises VE-Cadherin, zonula occludens-1 (ZO-1), occludin and claudin-5, which are central components of the TJs [4]. Loss of these membrane-associated adhesion molecules disrupts endothelial junction integrity and increases vascular permeability, consequently destroying the vascular barrier and facilitating metastasis of cancer cells [5–7].

Exosomes are nanosized vesicles (30–150 nm diameter) that are secreted by most cells. They are enclosed by a lipid bilayer and carry various biomolecules, including proteins, glycans, lipids, RNA, and DNA [8]. When exosomes are taken up by other cells, these cargoes are transferred and influence the phenotype of the recipient cells. As such, exosomes are viewed as essential mediators of cell-cell communication. To access distant tissues, cancer cell-secreted exosomes can impair endothelial cell junctions, which in turn leads to increased vascular permeability, promoting further entry of vesicles and cells into the tissue parenchyma [9]. Multiple studies have shown that exosomal RNAs and proteins induce vascular permeability. Gastric cancer-secreted exosomal X26nt increases angiogenesis and vascular permeability by targeting VE-cadherin [10]. Exosomes derived from hepatoma cells with high miR-103 expression increased the permeability of endothelial monolayers and the rates of hepatic and pulmonary metastases by directly inhibiting the expression of VE-Cadherin, p120-catenin and ZO-1 in endothelial cells [11]. Thrombospondin-1 was highly expressed in MDA-MB-231 breast cancer cell derived exosomes. Treating human umbilical microvascular endothelial cells (HUVECs) with Thrombospondin-1-enriched exosomes similarly promoted the transendothelial migration of malignant cells and decreased the expression of intercellular junction proteins [12]. Exosomes from breast cancer cells increase the permeability of the hCMEC/D3 cell layer via Tubulin Tyrosine Ligase Like 4 [13]. Exosomal VEGF-A derived from glioblastoma stem-like cells increases brain endothelial cell permeability and angiogenesis [14]. Collectively, these studies demonstrate that exosomes exploit various mechanisms to promote vascular leakiness in different cancer types.

Nasopharyngeal carcinoma (NPC) is a common malignant epithelial tumor of the nasopharynx. In 2018, there were ~129,000 new cases of NPC worldwide, and >70% of these new cases were in East and Southeast Asia [15]. According to the World Health Organization, type III NPC, which is nonkeratinizing and undifferentiated, is the most common subtype, as it accounts for 63-95% of all NPC cases worldwide [16, 17]. This type of NPC is invariably associated with Epstein-Barr virus (EBV) infection and has a higher incidence of distant metastasis [18]. Once NPC metastasizes distantly, a complete cure of metastatic NPC remains elusive even if the patients are treated with advanced therapies [19]. Thus, a full understanding of the molecular mechanism of NPC metastasis is essential.

There have been many studies on the contribution of EBV to the metastasis of NPC. Several EBV modulate processes related to cancer progression, such as epithelial-mesenchymal transition (EMT), cell motility and invasiveness. EBV drives NPC metastasis through EBV-encoded LMP1-mediated metabolic reprogramming via activation of IGF1-mTORC2 signaling and nuclear acetylation of the Snail promoter by PDHE1α [20], LMP1 upregulates calreticulin to induce EMT via the TGF-β/Smad3/NRP1 pathway in nasopharyngeal carcinoma cells [21]. LMP2A promotes the EMT in nasopharyngeal carcinoma via metastatic tumor antigen 1 and mechanistic target of rapamycin signaling induction [22]. LMP2A interacts with spleen tyrosine kinase and enhances the invasiveness and metastasis of NPC [23]. Research has also shown that EBNA1 upregulates the expression of its target genes, zinc finger E-box binding homeobox 1 (ZEB1) and ZEB2, which are well-known mediators of EMT [24]. In addition, EBV-encoded miRNAs also take part in NPC metastasis. For example, EBV-miR-BART1 and EBV-miR-BART7-3p directly downregulate the expression of PTEN [25, 26], which induces EMT and increases NPC migration, invasion and metastasis. EBV-miR-BART9 specifically represses VE-cadherin, which can induce the redistribution of β-catenin and promote the metastasis of NPC cells [27]. EBV-encoded circRPMS1 promotes nasopharyngeal carcinoma cell proliferation and metastasis through sponging multiple miRNAs [28]. However, much progress has been made in exploring the mechanisms that confer tumor cells with the ability to detach, migrate and invade, but less attention has been given to the mechanisms that regulate vascular permeability and thereby affect metastasis.

Herein, we showed that exosomes derived from EBV-positive NPC cells promote vascular permeability and EndMT. Furthermore, we demonstrate that exosomal HMGA2 protein mediates the formation of a premetastatic niche by inducing vascular leakiness and consequently promotes NPC metastasis. Finally, our clinical data suggest that HMGA2 from circulating exosomes of NPC patients may be used as a blood-based biomarker for the prediction of metastasis.

## Materials and methods

### Patient specimens

All blood samples (10 normal control, 10 NPC with metastasis and 42 NPC without metastasis) and tissue samples (10 normal and 26 NPC) of pathologically confirmed NPC patients were obtained from the General Hospital of Southern Theater Command, People’s Liberation Army of China. Informed consent was obtained from each patient and the protocol was approved by the Institutional Research Ethics Committee of the General Hospital of Southern Theater Command, People’s Liberation Army of China. Clinical stages were determined according to the China 2008 Guangzhou NPC staging system.

### Cell culture

HK1, NPC43, C666-1 and C17 cells were kindly provided by Professor S-W. Tsao, University of Hong Kong. HONE1, SUNE1, HNE1, CNE2, TW03 and 5-8F cells were provided by Professor Mu-Sheng Zeng, Sun Yat-sen University Cancer Center. The NPC cell lines were cultured in RPMI-1640 medium supplemented with 10% FBS (HyClone), 100 U ml^−1^ penicillin and 100 μg ml^−1^ streptomycin. The short tandem repeat of all the NPC cell lines were tested. HUVECs were purchased from ATCC (Rockefeller, Maryland, USA) and cultured in ECM (ATCC) supplemented with appropriate concentrations of growth factors. All cells were maintained at 37 °C with 5% CO_2_. All cell lines were routinely tested and shown to be mycoplasma-free as determined by the PCR-based method.

### Isolation and identification of exosomes derived from cultured cells

Exosomes were collected from cellular medium after ultracentrifugation as described previously [29]. To isolate exosomes, human NPC cell lines were cultured in a 15 cm dish with 30 ml of 10% exosome-depleted FBS in 1640 medium. The conditioned medium was collected 48 h later and centrifuged at 500 × *g* for 15 min to remove cells, 3000 × *g* for 15 min, and then at 12,000 × *g* for 30 min to remove cell debris. The supernatant was further ultracentrifuged at 110,000 × *g* for 70 min to collect exosomes at 4 °C. The pellets were washed by resuspension in PBS and ultracentrifuged at 110,000 × *g* for 70 min and then dissolved in RIPA (for protein isolation), PBS (for exosome identification and in vivo functional experiments) or 10% exosome-depleted FBS-ECM (for cell culture). The protein concentration of the harvested exosomes was detected by a bicinchoninic acid protein assay kit (Pierce, Thermo Scientific). Preparation of conditioned medium from transfected cells began 2 days after transfection to ensure optimal protein depletion in exosome-derived from cells. For transmission electron microscopy, exosomes were fixed with 2% glutaraldehyde and placed on 200-mesh formvar-coated grids. The grids were then stained using 2% phosphotungstic acid for 2 min and observed with a transmission electron microscope (Hitachi H-7500). Exosome size and particle number were analyzed using an NS300 (NanoSight, Malvern Instruments).

### Isolation and identification of exosomes derived from serum samples

As previously described [30], 3 ml of cell-free serum samples were thawed on ice. Serum was diluted in 132 ml PBS and filtered through a 0.2-μm pore filter, the samples were ultracentrifuged at 150,000 × *g* overnight at 4 °C. Next, the exosomes pellet was washed in 132 ml PBS followed by a second step of ultracentrifugation at 150,000 × *g* at 4 °C for 2 h. The supernatant was discarded and pelleted exosomes were resuspended in RIPA (for protein isolation) or 10% exosome-depleted FBS-ECM (for cell culture).

### Cellular internalization of exosomes

To prepare PKH67-labeled exosomes, exosomes were incubated with PKH67 (final concentration at 1 μM in PBS; Sigma-Aldrich, St. Louis, MO) for 10 min, followed by serum neutralization and two washes in PBS at 110,000 × *g* for 70 min to remove the excess PKH67. The pellets were then resuspended in 10% exosome-depleted FBS-ECM, added to the HUVECs at 80% confluence at concentration of 5 μg ml^−1^, and incubated for 2 h before imaging under a fluorescence microscope.

### Antibodies

The following antibodies were used in this study: rabbit polyclonal ZO-1 antibody (abcam, ab96587), rabbit polyclonal VE-cadherin (abcam, ab232880, ab33168), Rabbit monoclonal Occludin (abcam, ab216327), Rabbit monoclonal Claudin-5 (abcam, ab131259), Rabbit polyclonal CD31 (abcam, ab124432, ab9498), Rabbit monoclonal α-SMA (Cell Signaling Technology, 19245), Rabbit monoclonal Vimentin (abcam, ab92547), Rabbit monoclonal S100A4 (abcam, ab197896), Rabbit polyclonal HMGA2 (abcam ab97276), Rabbit polyclonal Snail (abclonal, A11794), Rabbit monoclonal Syntenin (abcam, ab133267), Rabbit monoclonal CD63 (abcam, ab134045), Rabbit polyclonal Calnein (abcam, ab75801), Mouse monoclonal VDAC1 (abcam, ab14734), Rabbit monoclonal DDX6 (abclonal, A9634), Rabbit polyclonal CEMIP (abclonal, A8587), Rabbit monoclonal CEMIP (abclonal, A9664), Rabbit polyclonal MTA2 (abclonal, A2243), Mouse monoclonal β-Actin antibody (A5441, Sigma).

### Western blotting

For Western blotting, the cell lysates were separated in SDS-polyacrylamide gels, electrophoretically transferred to a nitrocellulose membrane (Milipore), and incubated sequentially with primary and secondary antibodies. Signals were detected with chemiluminescence reagents (ECL kit, Pierce Biotechnology) and imaged with a Universal Hood II Chemiluminescent Imaging System (Bio-Rad, California, USA).

### Immunofluorescence staining

For immunofluorescence staining of cells, HUVECs (3 × 10^4^) were seeded on a cover glass in a 24-well plate and cultured with medium without or with exosomes (at a concentration of 5 μg ml^−1^) for 48 h, fixed with 4% paraformaldehyde and then incubated with primary antibodies for 12 h at 4 °C. The cells were then incubated with Alexa Fluor-conjugated secondary antibody (Life Technologies) at room temperature for 1 h. The nuclei were counterstained with 4-diamidino-2-phenylindole (DAPI, Sigma-Aldrich). Immunofluorescence images were then obtained under a fluorescence microscope. For immunofluorescence staining of tissue sections, paraffin sections were subjected to antigen retrieval with citrate and washed twice with PBS. Blocking buffer (10% goat serum in PBS) was added for 30 min, and samples were then stained with primary antibodies for 12 h at 4 °C and goat anti-rabbit IgG/Alexa Fluor or goat anti-rat IgG/Alexa Fluor for 1 h at room temperature, counterstained with DAPI before imaging under a fluorescence microscope.

### Immunohistochemistry (IHC)

IHC was performed on formalin-fixed paraffin-embedded sections of clinical NPC tissues and mouse xenograft tissues. Briefly, the tissues were deparaffinized and rehydrated, subjected to citrate-mediated high-temperature antigen retrieval, and incubated with primary antibodies at 4 °C overnight. The stained slides were scored according to the intensity of staining (−: 0; +: 1; ++: 2; and +++: 3) and the percentage of the cells of interest staining positive for each antigen (0%: 0; 1~29%: 1; 30~69%: 2; and ≥70%: 3). The intensity score was multiplied by the percentage score to obtain a final score, which was used in statistical analyses. The investigator was blinded to the group allocation.

### In vitro endothelial permeability assay

In vitro endothelial permeability was assessed by quantifying the amount of rhodamine B isothiocyanate-dextran (rhodamine-dextran, average MW ~70,000; Sigma-Aldrich) that passed through the endothelial monolayers. Briefly, endothelial cells (2 × 10^4^) were plated on a 24-well Transwell filter (0.4-μm pore size; Corning), and 10% exosome-depleted FBS-1640 medium with exosomes derived from NPC cells with or without EBV at the concentration of 5 μg ml^−1^ was added into both upper and lower chambers, and then incubated for 48 h to allow the cells to reach 100% confluence. Rhodamine-dextran was then added to the upper chamber at 20 mg ml^−1^, and 1 h later, the amount of rhodamine-dextran in the lower chamber was determined by measuring the fluorescence intensity using a microplate spectrofluorometer (BioTek, Vermont, USA) at a 544 nm excitation and a 590 nm emission wavelength. The investigator was blinded to the group allocation.

### Transendothelial invasion assay

HUVECs (2 × 10^4^) were plated on 24-well Transwell filters (3-μm pore size; Corning), and NPC cell-derived exosomes (in 10% exosome-depleted FBS-1640 medium at a concentration of 5 μg ml^−1^) were added to both the upper and lower chambers, and then incubated for 48 h to allow the cells to reach 100% confluence. The medium in both the upper and lower chambers was then removed, and 5 × 10^4^ GFP-expressing 5-8F cells in 100 μl of serum-free 1640 medium were added to the upper chamber, while the lower chamber was filled with 600 μl of 10% exosome-depleted FBS-1640 medium. After incubation for 24 h, cells remaining on the upper surface of the filter were scraped off. The GFP-expressing NPC cells that invaded through the HUVEC monolayer to the lower surface of the filters were fixed and then counted under a fluorescence microscope. The investigator was blinded to the group allocation.

### Lentivirus mediated overexpression and knockdown

Lentiviruses for overexpression and knockdown were provided by Vigene Biosciences (Jinan, Shandong). To establish HUVEC and HK1 sublines that stably expressed CD82, CEMIP, DDX6, MTA2 and HMGA2, NPC43 sublines that stably expressed low levels of CD82, CEMIP, DDX6, MTA2 and HMGA2 and their matched control lines, lentivirus was added to medium supplemented with 10 µg ml^−1^ polybrene (Sigma-Aldrich) and then the medium was directly incubated with HK1 cells, HUVECs and NPC43 cells. GFP-expressing cells were sorted by flow cytometry (Beckman Coulter, Brea, CA). The overexpression and knockdown efficiency were determined by Western blotting.

### Mass spectrometry

Mass spectrometry analyses of exosomes from HK1 and NPC43 cells were performed at Shanghai Applied Protein Technology as previously reported [31].

### Animals

Eight-week-old male athymic BALB/c-nu mice were purchased from Beijing Vital River Laboratory Animal Technology Company (Beijing, China) and maintained under specific pathogen-free conditions. All protocols for animal studies were reviewed and approved by the Institutional Animal Care and Use Committee of Southern Medical University. For education experiments, mice (six mice per group, mice of each group were chosen randomly) were injected with 5 µg exosomes via the tail vein every other day for 2 weeks. Exosome-treated mice were either killed for tissue collection and TJ protein alteration assessment or subjected to injection of rhodamine-dextran or NPC cells. For the in vivo permeability assay, rhodamine-dextran was intravenously injected into the tail vein of nude mice 2 h before transcardiac perfusion was carried out to remove the excess dye, the mouse livers and lungs were enucleated for examination. For the metastasis assay, 2 × 10^6^ NPC cells were injected into the tail vein of exosome-treated nude mice. After 45 days, mice injected with NPC cells were sacrificed, and the lungs and livers were removed for examination. For the liver metastasis model, under anesthesia with a peritoneal injection of 1% pentobarbital sodium, a small cut was made in the abdominal region, the mouse liver was gently pushed out of the abdominal cavity, and a 1 mm^3^ piece of xenograft tumor derived from 5-8F (2 × 10^6^) cells with or without HMGA2 overexpression was injected into the liver tissue (six mice per group). All mice were killed when the liver tumor fluorescence intensity of control group is no significant difference with that of the HMGA2 overexpressing group. Tumor tissues were fixed in 4% paraformaldehyde for 24 h and transferred to gradient ethanol solutions. Tumors were embedded in paraffin, sectioned with a Leica RM2235 and processed for histological examination. The investigator was blinded to the group allocation.

### Statistical analysis

Data are expressed as the mean ± SD after analysis by SPSS 14.0 software. When the population could not be assumed to be normally distributed, Student’s *t* test was used for statistical analysis of data from two groups, and Dunnett’s test was used for statistical analysis of data from more than two groups. *P < 0.05, **P < 0.01 and ***P < 0.001 indicate statistical significance.

## Results

### Exosomes derived from EBV-positive NPC cells destroy barrier function and promote EndMT in endothelial monolayers

**Fig. 1:**
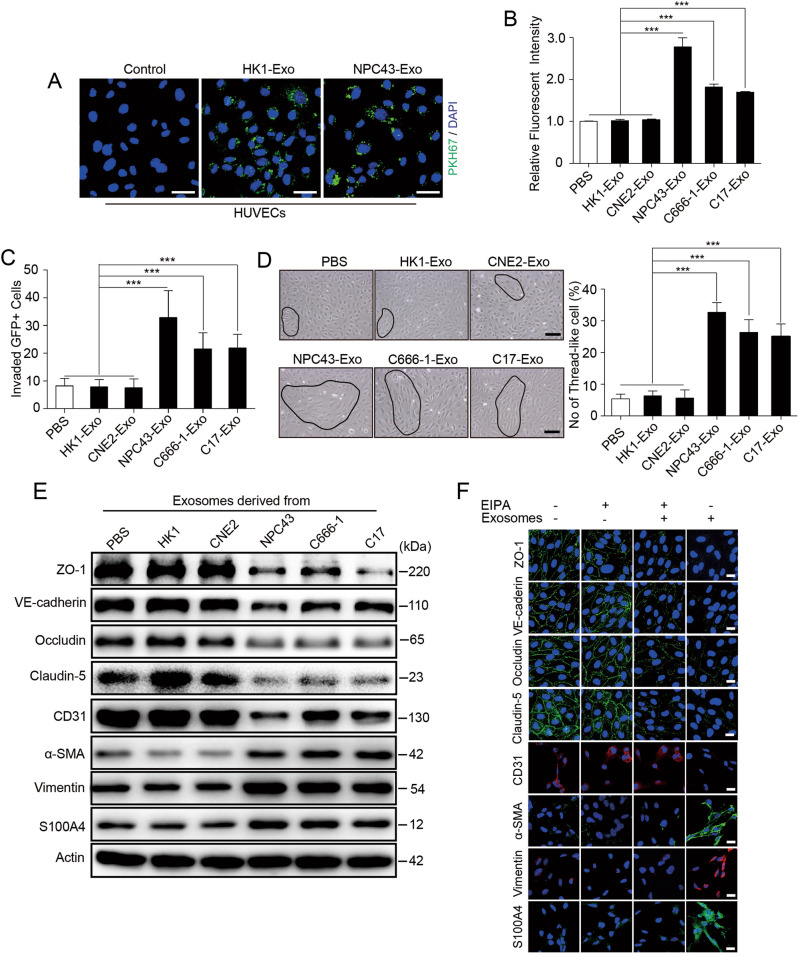
Exosomes derived from EBV-positive NPC cells reduce endothelial cell TJ protein expression and promote EndMT. **A** Confocal microscopy analysis of HUVECs treated with PKH-67 (green)-labeled purified NPC cell-derived exosomes or PKH-67 wash buffer (scale bar = 20 μm). **B** The permeability of treated HUVEC monolayers grown on 0.4 μm filters was measured by the appearance of rhodamine-dextran, which was added to the top well at the beginning of the experiment, in the bottom well during a 2 h time course. The absorbance at 590 nm at each time point is indicated. ***p < 0.001. **C** HUVEC monolayers grown on 3 μm filters were treated as indicated before GFP-labeled 5-8F cells were seeded in the Transwell inserts. After 10 h, the GFP+ cells on the bottom side of the filters were quantified under a fluorescence microscope. ***p < 0.001. **D** Representative images (left panel) and histogram of the quantification (right panel) of endothelial cellular morphology alterations after the cells were treated with PBS, HK1-Exo, CNE2-Exo, NPC43-Exo, C666-1-Exo and C17-Exo. ***p < 0.001. **E** HUVECs treated with PBS, HK1-Exo, CNE2-Exo, NPC43-Exo, C666-1-Exo and C17-Exo were analyzed by Western blotting for TJ proteins (ZO-1, VE-cadherin, occludin and claudin-5), CD31, α-SMA, Vimentin and S100A4. **F** HUVEC monolayers were treated as indicated for 48 h and analyzed by immunofluorescence for ZO-1 (green), VE-cadherin (green), occludin (green) and claudin-5 (green), CD31 (red), α-SMA (green), Vimentin (red) and S100A4 (green). DAPI (blue): cell nuclei.

We chose EBV-positive NPC cell lines and EBV-negative NPC cell lines as models for studying cancer-secreted exosomes. Exosomes purified from conditioned medium by ultracentrifugation exhibited the typical cup-shaped morphology by electron microscopy (Supplementary Fig. S1A) and had a size range of 50–200 nm (Supplementary Fig. S1B). Additionally, Western blot analysis revealed that the vesicles were positive for the exosome markers syntenin and CD63 but negative for endoplasmic reticulum calnexin and mitochondrial VDAC1 (Supplementary Fig. S1C). We focused on endothelial cells in this study because of their critical barrier function during metastasis. When exosomes labeled with the fluorescent dye PKH67 were incubated with primary HUVECs, the recipient cells exhibited high uptake efficiency, as indicated by fluorescence microscopy (Fig. 1A). To explore the function of exosomes derived from EBV-positive NPC cells on endothelial cells, endothelial cells were treated with PBS or exosomes from EBV-negative cells (HK1 and CNE2) and EBV-positive NPC cells (NPC43, C666-1 and C17). We performed an in vitro permeability assay by measuring the traversal of rhodamine-labeled dextran (relative molecular mass 70,000) probes through HUVEC monolayers growing on 0.4 μm filters. Pretreatment with exosomes derived from EBV-positive NPC cells allowed much more dextran to traverse through the HUVEC monolayer, suggesting increased endothelial permeability. However, treatment with exosomes from EBV-negative NPC cells or PBS increased the transendothelial transport of dextran only marginally (Fig. 1B). Next, to directly simulate the barrier-traversal step of metastasis, transendothelial invasion of cancer cells was examined using HUVEC monolayers grown on 3 μm filters. The number of GFP-labeled 5-8F cells that had invaded through HUVECs treated with exosomes derived from EBV-positive NPC cells was significantly greater than that of cells that had invaded through HUVECs treated with PBS or exosomes derived from EBV-negative NPC cells (Fig. 1C). Strikingly, the morphology of monolayer cells treated with exosomes from EBV-positive NPC cells switch from a pebble-shaped to a spindle-shaped morphology (Fig. 1D). We next examined the changes in the central molecular components of TJs (ZO-1, VE-cadherin, occludin and claudin-5), which comprise the major group of cell-cell adhesion complexes in endothelial cells. Treatment with exosomes derived from EBV-positive NPC cells but not EBV-negative NPC cells, resulted in a significant decrease in TJ protein levels in HUVECs, accompanied by a marked decrease in endothelial markers such as cluster of differentiation 31 (CD31) and an increased expression of mesenchymal biomarkers such as α-SMA, Vimentin, and S100A4 (Fig. 1E). Consistent with the above results, treatment with EIPA, an exosome uptake inhibitor [32], reversed the morphological changes in endothelial cells induced by NPC43 exosomes (Supplementary Fig. S2A). EIPA also significantly decreased the traversal of rhodamine-labeled dextran (Supplementary Fig. S2B), inhibited the reduction in TJ proteins levels (Fig. 1F and Supplementary Fig. S2C) and increased mesenchymal markers expression in HUVECs (Fig. 1I and Supplementary Fig. S2D), further supporting the association of exosomes derived from EBV-positive NPC cell lines with increased endothelial cell permeability and EndMT. Taken together, our data proved that exosomes derived from EBV-positive NPC cells downregulate endothelial cell TJ protein expression, destroy barrier function and promote EndMT in vitro.

### Exosomes derived from EBV-positive NPC cells induce vascular permeability and promote metastasis in vivo

**Fig. 2:**
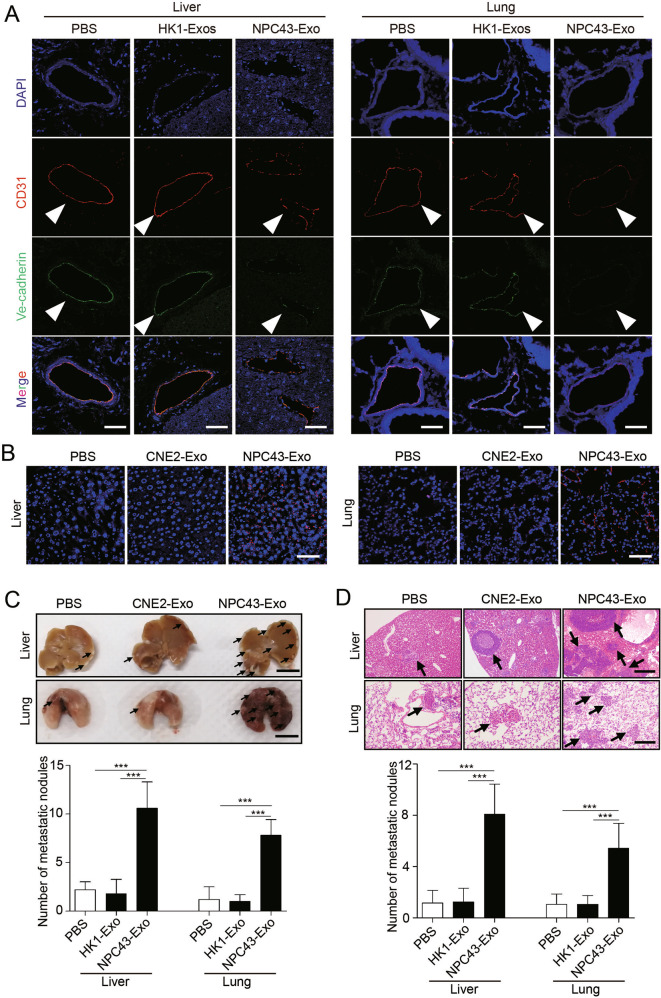
Exosomes derived from EBV-positive NPC cells induce vascular permeability and promote metastasis in vivo. **A** Collected lung and liver tissues were subjected to double-label immunofluorescence for VE-cadherin (green) and CD31 (red). Structures positive for CD31 are indicated by arrowheads. The scale bar represents 100 μm. **B** In vivo vascular permeability determined by the appearance of intravenously injected rhodamine-dextran in tissue (red) (*n* = 6). Representative images are shown. DAPI (blue): cell nuclei. The scale bar represents 100 μm. **C** Gross appearance of the liver and lung in the tail vein injection metastatic model and metastatic lesion quantitative analysis. Data are presented as the mean ± SD (*n* = 6 biologically independent animals). ***p < 0.001. **D** Representative hematoxylin and eosin (H&E) stained metastatic tumor nodules in the liver and lung, and quantitative analysis of metastatic nodules. Data are presented as the mean ± SD (*n* = 6 biologically independent animals). ***p < 0.001.

To further demonstrate the in vivo effect of NPC cell-derived exosomes, we injected exosomes secreted by HK1 cells (an EBV-negative NPC cell line), exosomes secreted by NPC43 cells (an EBV-positive NPC cell line), or PBS as a control into the tail veins of Balb/c null mice and examined the lung and liver, organs that frequently host NPC metastases, after exosome treatment. To assess lung and liver exosome distribution, PKH67-labeled exosomes were injected via the tail vein. The endothelial cell membrane (CD31 positive) colocalized with green PKH67-labeled exosomes, indicating that endothelial cells can take up exosomes in vivo (Supplementary Fig. S3). Compared with that in the PBS and HK1 exosome-treated groups, ZO-1, VE-cadherin, occludin and claudin-5 expression were significantly reduced in endothelial cells positive for CD31 (Fig. 2A and Supplementary Fig. S4), and much more dextran extravasated from the blood circulation into the interstitial space of lung and liver in NPC43 exosome-treated group (Fig. 2B), indicating that the vasculature in mice treated with exosomes derived from EBV-positive NPC cells exhibited higher permeability. To confirm whether vascular permeability induced by exosomes from EBV-positive NPC cells promotes NPC metastasis, NPC43 cells were injected into the tail veins of nude mice pretreated with exosomes derived from HK1 cells and NPC43 cells or PBS. As expected, there were more hepatic and pulmonary metastatic colonies in mice injected with exosomes from NPC43 cells than in those injected with exosomes from HK1 cells or PBS (Fig. 2C, D). These in vivo data support the conclusion that exosomes from EBV-positive NPC cells increase vascular permeability and consequently promote tumor metastasis.

### Exosomal HMGA2 protein from EBV-positive NPC cells mediates vascular permeability increase and EndMT

**Fig. 3:**
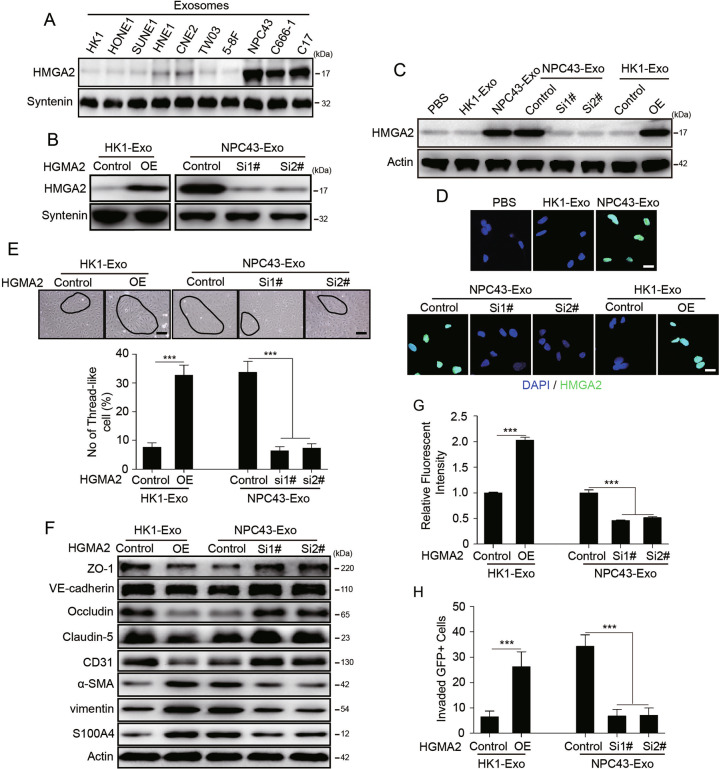
HMGA2 is enriched in exosomes derived from EBV-positive NPC cells and increases vascular permeability and EndMT in endothelial cells in vitro. **A** HMGA2 protein levels in exosomes derived from EBV-negative NPC cell lines (HK1, HONE1, SUNE1, HNE1, CNE2, TW03 and 5-8F cells) and EBV-positive NPC cell lines (NPC43, C666-1 and C17 cells) were detected by Western blotting. Western blotting was performed at least three times. **B** Analysis of the HMGA2 content in corresponding cell-derived exosomes after overexpression and knockdown of HMGA2 in HK1 cells and NPC43 cells respectively. The change in HMGA2 expression in HUVECs treated as indicated for 12 h was analyzed by Western blotting (**C**) and immunofluorescence for HMGA2 (green), with DAPI (blue) staining of cell nuclei (**D**). Western blotting was conducted three times. **E** Representative images (high panel) and histogram of the quantification (low panel) of the endothelial cellular morphology alteration after the cells were treated as indicated for 48 h. ***p < 0.001. **F** HUVECs treated as indicated for 48 h were analyzed by Western blotting for the TJ proteins, CD31, α-SMA, Vimentin and S100A4. Western blotting was conducted three times. **G** The permeability of treated HUVEC monolayers grown on 0.4 μm filters was measured by the appearance of rhodamine-dextran. ***p < 0.001. **H** HUVEC monolayers grown on 3 μm filters were treated as indicated before GFP-labeled 5-8F cells were seeded in the Transwell inserts. After 10 h, the GFP+ cells on the bottom side of the filters were quantified under a fluorescence microscope. ***p < 0.001.

Previous reports have shown that tumor exosomes package specific proteins critical for the metastatic process. To determine how exosomes from EBV-positive NPC cells regulate vascular permeability, quantitative mass spectrometry was performed to compare the exosome proteome from NPC43 cells to that of HK1 cells. Because only exosomes from EBV-positive NPC cells increase endothelial cell permeability and EndMT, we looked for proteins that were enriched in NPC43-derived exosomes compared to HK1-derived exosomes. Analysis of the data revealed that 181 proteins were enriched >2-fold in NPC43 cell exosomes compared to HK1 cell exosomes (Supplementary Fig. S5A). Further analysis of these 181 proteins using KEGG revealed enrichment in protein classes, including regulation of EMT, the actin cytoskeleton, focal adhesion and TJs (Supplementary Fig. S5B). Based on this analysis, 19 out of the 181 proteins were related to the above functions (Supplementary Fig. S5C). We prioritized these 19 proteins as top candidates for promoting increases in vascular permeability and EndMT. Among these proteins, HMGA2, CEMIP, DDX6, MTA2 and CD82 emerged as prominent proteins in exosomes from all three EBV-positive NPC cell lines and had low or undetectable expression in exosomes from seven EBV-negative NPC cell lines (Fig. 3A and Supplementary Fig. S6A), suggesting a specific association with vascular destruction potential and EndMT. To identify the function of HMGA2, CEMIP, DDX6, MTA2 and CD82 in endothelial cell permeability and EndMT, we overexpressed the above five proteins in HUVECs (Supplementary Fig. S6B) and then examined the morphological change of endothelial cells and permeability by measuring the traversal of rhodamine-labeled dextran probes through HUVEC monolayers. The results showed that only overexpression of HMGA2 can switch the morphology of endothelial cells from pebble-shaped to spindle-shaped (Supplementary Fig. S6C) and increase vascular permeability (Supplementary Fig. S6D), indicating that HMGA2 may account for endothelial cell functional changes induced by exosomes derived from EBV-positive NPC cells.

To test the function of exosomal HMGA2 in vascular permeability, we overexpressed HMGA2 in HK1 cells and targeted HMGA2 in NPC43 cells using shRNA. As expected, HMGA2 content was increased in HMGA2-overexpressing HK1 cell-derived exosomes, whereas the reduction in HMGA2 expression in NPC43 cells had the opposite effect (Fig. 3B). Transmission electron microscopy (Supplementary Fig. S7A) and nanoparticle tracking analysis (Supplementary Fig. S7B) revealed that changes in HMGA2 did not affect exosome morphology or size. In addition, the particles/protein ratio (Supplementary Fig. S7C) and expression of CD63, Syntenin-1, Calnexin and VDAC1 (Supplementary Fig. S7D) remained unaltered in exosomes derived from HMGA2 KD NPC43 cells and exosomes from HMGA2-overexpressing HK1 cells, suggesting that HMGA2 does not alter exosome secretion and exosomal protein packaging.

We found that HMGA2 expression was significantly higher in EBV-positive NPC cells than in endothelial cells (Supplementary Fig. S8A), indicating that the background level of HMGA2 in HUVECs was low. Interestingly, the HMGA2 protein level in HUVECs was increased upon treatment with NPC43 cell derived exosomes (Fig. 3C), which is primarily located in the nucleus where its function as a transtriptional factor (Fig. 3D). Furthermore, the mRNA level of HMGA2 in HUVECs treated with EBV-positive NPC cell derived exosomes was unchanged (Supplementary Fig. S8B), so we excluded the possibility that HMGA2 protein level was increased due to endogenous synthesis or transfer of HMGA2 mRNA.

Decreasing HMGA2 level in NPC43-derived exosomes reduced the ability of exosomes to induce morphological changes. However, increased HMGA2 in exosomes from HK1 cells had the opposite effects (Fig. 3E). Western blot analysis showed that exosomes carrying HMGA2 can reduce the TJ protein and endothelial cell marker expression and increase mesenchymal cell marker expression (Fig. 3F). Furthermore, a reduction of HMGA2 levels in NPC43 cell-derived exosomes significantly impaired the association of exosome treatment with vascular permeability, with a 50% reduction in passage of the fluorescent probes and a 75% reduction in invading cancer cells compared to those observed after treatment with exosomes derived from control cells. Consistent with these metrics, overexpression of HMGA2 in HK1 cell-derived exosomes promoted the passage of fluorescent probes, and cancer cells invaded the endothelial cell monolayer (Fig. 3G, H). Taken together, our data show that HMGA2, a protein enriched in exosomes from EBV-positive NPC cell lines, promotes increases in endothelial cell permeability and EndMT.

### Snail-mediated HMGA2 induces endothelial cell permeability and EndMT

**Fig. 4:**
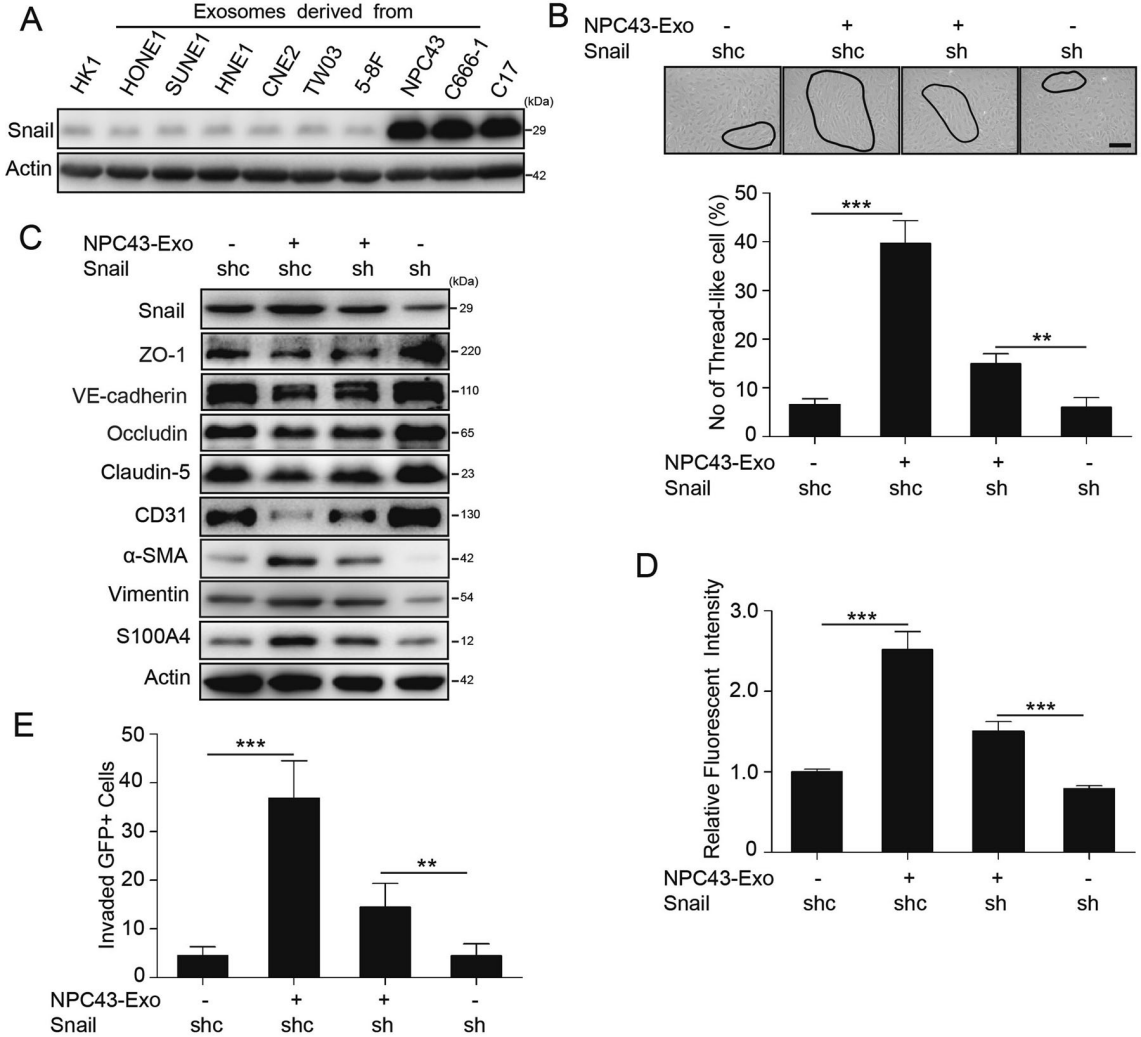
Exosomal HMGA2 reduces endothelial cell TJ protein expression and promotes EndMT via Snail. **A** Top, pathways affected by NPC43 cells derived exosomes in HUVECs compared with HK1 cells derived exosomes and PBS. Bottom, a heatmap of differentially expressed genes involved in selected pathways. **B** Top, pathways affected by HMGA2 in HUVECs with HMGA2 overexpression compared with the control. Bottom, a heatmap of differentially expressed genes involved in selected pathways. **C** Western blot analysis of Snail level in HUVECs following treatment with exosomes derived from EBV-negative NPC cells (HK1, HONE1, SUNE1, HNE1, CNE2, TW03 and 5-8F cells) and EBV-positive NPC cells (NPC43, C666-1 and C17 cells). Western blot was conducted three times. **D** Representative images (high panel) and histogram of the quantification (low panel) of the endothelial cellular morphology alteration after the cells were treated as indicated for 48 h. ***p < 0.001. **E** HUVECs were infected with shSnail for 48 h and then treated with NPC43-Exo for 48 h. The cell lysate was analyzed by Western blotting for the TJ proteins, CD31, α-SMA, Vimentin and S100A4. Western blotting was conducted three times. **F** The permeability of HUVEC monolayers treated as indicated was measured by the absorption value of rhodamine-dextran. ***p < 0.001. **G** HUVEC monolayers grown on 3 μm filters were treated as indicated before GFP-labeled 5-8F cells were seeded in the Transwell inserts. After 10 h, the GFP+ cells on the bottom side of filter were quantified under a fluorescence microscope. ***p < 0.001.

Since our findings suggest a critical role for exosomal HMGA2 in endothelial cell permeability, we sought to identify mechanism involved in this process. To identify genes modulated by exosomal HMGA2, we first focused on genes significantly altered in endothelial cells by NPC43 cell-derived exosomes compared to HK1 cell-derived exosomes and PBS control, and then on the genes that showed significant and concordant differences in expression when compared HUVECs with HMGA2 overexpression and control HUVECs, we found 94 common genes with altered expression in endothelial cells in above two groups. Gene ontology analysis identified cell adhesion and EMT as two significantly affected processes in both groups, and CLDN3, ID1, CYFIP3, HLX3, TGM2, HGF, IL6 and TGFβ were the common changed genes in cell adhesion junction and EMT, which was in accordance with the results from single-cell transcriptomic analysis of endothelial cells in EBV positive NPC specimens [33] (Supplementary Figs. S9A and S9B). Both EMT and EndMT give rise to cells that have a similar mesenchymal phenotype, and current evidence suggests that both processes utilize common signaling pathways [34, 35], EMT has been extensively studied and has provided a useful framework to guide research on EndMT, and EndMT also can induce loss of tight junction and reduced cell adhesion in ECs [36], so we inferred HMGA2 may contribute to EndMT. Ectopic expression of HMGA2 in epithelial cells induces EMT via Snail, Twist and Slug, we speculated that HMGA2 may promote EndMT signaling similar to EMT. Western blot analysis confirmed that exosomes from EBV-positive NPC cells could increase only Snail expression (Fig. 4A). Knockdown of Snail in HUVECs offset the effect of NPC43 exosomes on cellular morphology (Fig. 4B), reduced TJ protein expression, increased mesenchymal cell marker expression (Fig. 4C), and promote the traversal of rhodamine-labeled dextran (Fig. 4D) and the transendothelial invasion of cancer cells (Fig. 4E). Collectively, these results suggest that HMGA2 induces endothelial cell permeability and EndMT via Snail.

### Exosomal HMGA2 promotes vascular permeability and NPC metastasis in vivo

**Fig. 5:**
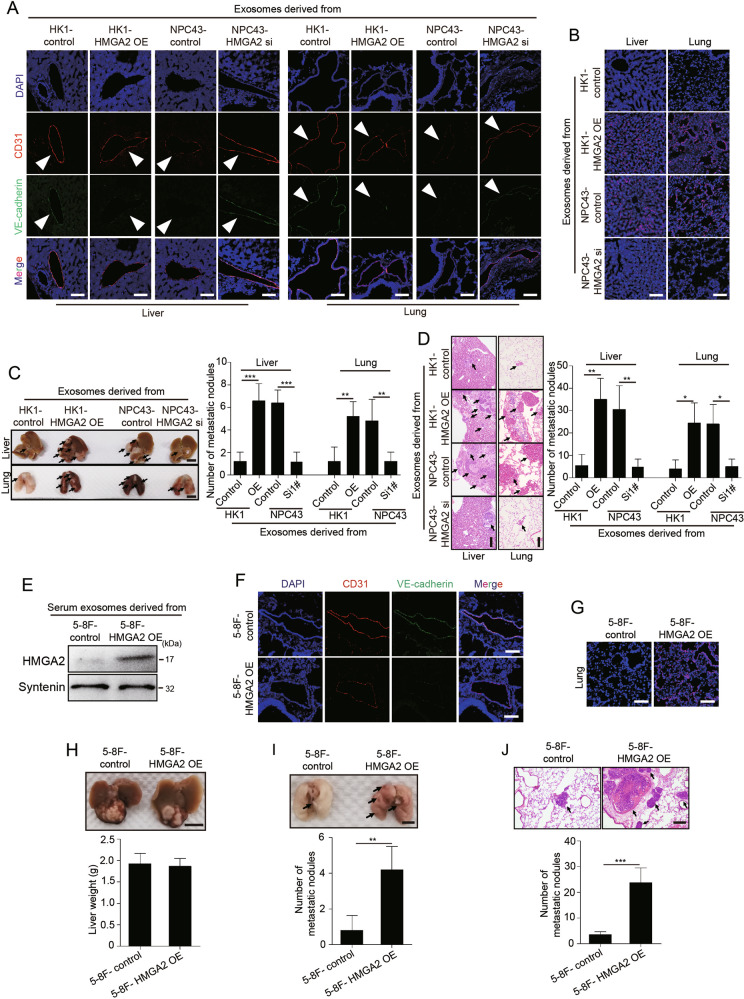
Exosomal HMGA2 derived from EBV-positive NPC cells induces vascular permeability and promotes metastasis in vivo. **A** BALB/C nude mice were pretreated with exosomes derived from the indicated cells every two days for four times, and then the collected lung and liver tissues were subjected to double-label immunofluorescence for VE-cadherin (green) and CD31 (red). Structures positive for CD31 are indicated by arrowheads. The scale bar represents 100 μm. **B** The vasculature exhibited higher permeability after treatment by HMGA2-overexpressing exosomes. Mice pretreated with exosomes with high or low HMGA2 expression were intravenously injected with rhodamine-dextran, and frozen tumor tissue sections were used for imaging by fluorescence microscopy. Rhodamine-dextran exhibited red fluorescence and cell nuclei were stained blue with DAPI. Scale bar, 50 μm. **C** Gross appearance of the liver and lung in the tail vein injection metastatic model and metastatic lesion quantitative analysis. Data are presented as the mean ± SD (*n* = 6 biologically independent animals). ***p < 0.001. **D** Representative H&E-stained metastatic tumor nodules in the liver and lung and quantitative analysis of metastatic nodules. Data are presented as mean ± SD (*n* = 6 biologically independent animals). ***p < 0.001. **E** The expression of HMGA2 in mouse serum-derived exosomes from mice bearing tumors derived from 5-8F cells with and without HMGA2 overexpression. **F** The tumor mass of subcutaneous xenograft tumors derived from of 5-8F cells with and without HMGA2 overexpression was transplanted into the liver of BALB/C nude mice. Collected lungs were subjected to double-label immunofluorescence for ZO-1 (green) and CD31 (red). Structures positive for CD31 are indicated by arrowheads. The scale bar represents 100 μm. **G** The lung vasculature permeability in BALB/C nude mice bearing 5-8F HMGA2 OE control or 5-8F HMGA2 OE tumors was measured by rhodamine-dextran in lung tissue. Rhodamine-dextran exhibited red fluorescence and cell nuclei were stained blue with DAPI. Scale bar, 50 μm. **H** 5-8F/vec or 5-8F/HMGA2 tumors were injected into the liver, and gross appearance of the liver and liver weight quantitative analysis in different groups are shown. **I** Gross appearance of the lung in the liver metastatic model and metastatic lesion quantitative analysis. Data are presented as the mean ± SD (*n* = 6 biologically independent animals). ***p < 0.001. **J** Quantitative analysis of representative H&E-stained the number of metastatic tumor nodules in the lung and metastatic nodules. Data are presented as the mean ± SD (*n* = 6 biologically independent animals). ***p < 0.001.

To further demonstrate the in vivo effect of exosomal HMGA2 on endothelial barriers, we injected exosomes secreted by HK1 cells/vec (low-HMGA2), HK1 cells/HMGA2 OE (overexpression) (high-HMGA2), NPC43/vec (high-HMGA2) cells, or NPC43/HMGA2 sh (low-HMGA2) into the tail veins of Balb/c null mice and examined the lungs and livers. The results showed that exosomes with high HMGA2 levels, but not those with low HMGA2 levels, significantly reduced ZO-1, VE-cadherin, occludin and claudin-5 expression in endothelial cells positive for CD31 (Fig. 5A and Supplementary Fig. S10) and enhanced vascular permeability (Fig. 5B). Furthermore, mice were pretreated with exosomes secreted by HK1 cells/vec, HK1 cells/HMGA2 OE, NPC43/vec cells, or NPC43/HMGA2 sh before tail vein injection of NPC43 cells. Four weeks later, the liver and lung tissues were collected for analysis. Mice pretreated with exosomes from HK1 cells/HMGA2 OE and NPC43/vec cells displayed higher rates of hepatic and lung metastases and also showed more and larger metastatic nodules in both the liver and lung (Fig. 5C, D).

To determine whether the HMGA2 level in primary tumors regulates endothelial barriers and metastasis, we stably overexpressed HMGA2 in 5-8F cells. Tumor lumps derived from subcutaneous tumors derived from 5-8F control or 5-8F HMGA2 OE cells were transferred into the livers of nude mice. Exosomes derived from the serum of mice bearing HMGA2-overexpressing tumors had more abundant HMGA2 than exosomes derived from the control group (Fig. 5E). Reduced levels of ZO-1, VE-cadherin, occludin and claudin-5 were observed in CD31-positive vascular endothelial cells in the lungs of mice with high-HMGA2 xenografts (Fig. 5F and Supplementary Fig. S11). In addition, the in vivo vascular permeability in the lungs of mice bearing HMGA2-overexpressing tumors was dramatically increased compared with that in the control group (Fig. 5G). To rule out the effect of tumor growth on metastasis, lungs were enucleated when the livers bearing 5-8F control tumors reached the same size as the lives bearing HMGA2-overexpressing tumors (Fig. 5H). Distant metastases were significantly induced in the lungs of mice bearing HMGA2-overexpressing (Fig. 5I, J). Collectively, these results suggest that tumor cells expressing and consequently secreting higher levels of HMGA2 acquire greater metastatic potential through weakened endothelial barriers in vivo.

### HMGA2 is a serum biomarker for metastatic progression and is associated with endothelial cell TJ protein expression and EndMT in NPC

**Fig. 6:**
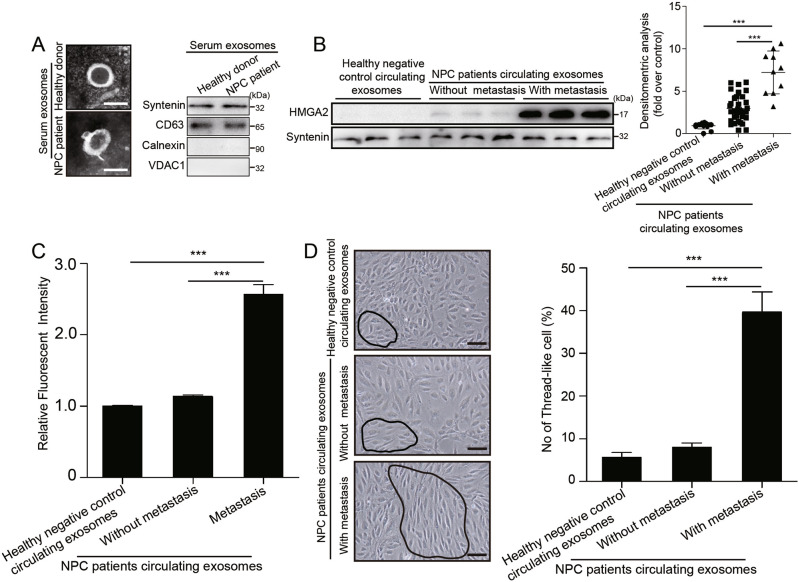
HMGA2 is associated with metastatic progression, TJ protein expression and EndMT in NPC. **A** The characteristics of healthy negative control serum exosomes and NPC serum exosomes were identified by transmission electron microscopy and Western blotting. **B** The content of HMGA2 in healthy negative control serum exosomes and NPC serum exosomes with and without metastasis. Densitometry was performed with ImageJ. ***p < 0.001. **C** The permeability of HUVEC monolayers treated with circulating exosomes from healthy negative controls and circulating exosomes from NPC patients with and without metastasis was measured by the absorption value of rhodamine-dextran. ***p < 0.001. **D** Representative images (left panel) and histogram of the quantification (right panel) of the endothelial cellular morphology alteration after the cells were treated with serum exosomes from healthy negative controls and serum exosomes from NPC patients with and without metastasis for 48 h. ***p < 0.001.

Because HMGA2 is expressed and secreted by NPC cells, it is possible that cancer-secreted HMGA2 can be detected in the circulation of NPC patients, where HMGA2 may serve as a prognostic marker for metastatic potential. To explore this possibility, we determined that the level of exosomal HMGA2 in the serum correlated with metastasis. Circulating exosomes were extracted from serum of the healthy donors and NPC patients with or without metastasis. Transmission electron microscopy and Western blotting showed that there was no significant difference between healthy donor serum-derived exosomes and NPC patient serum-derived exosomes (Fig. 6A). Western blot analysis showed that the HMGA2 level of circulating exosomes was increased in NPC patients compared with healthy donors. Moreover, the HMGA2 level in circulating exosomes from NPC patients with metastasis was higher than those in circulating exosomes from NPC patients without metastasis (Fig. 6B). To further determine whether circulating HMGA2 in NPC patients is functionally active in regulating endothelial cells, we treated HUVECs with exosomes from serum of a healthy donor (low HMGA2) or NPC patients with (high HMGA2) and without metastasis (moderate HMGA2). Exosomes derived from serum of the patients with metastatic but not the normal control serum or serum of the patients without metastatic increased vascular permeability (Fig. 6C) and promoted the switch from a pebble-shaped to a spindle-shaped morphology of endothelial cell (Fig. 6D). In addition, IHC staining of HMGA2, Snail, CD31, TJ proteins, α-SMA, Vimentin and S100A4 in a series of sections of primary NPC specimens showed that the expression of HMGA2 in tumors was negatively correlated with CD31 and TJ protein expression but positively correlated with Snail, α-SMA, Vimentin and S100A4 expression in endothelial cells (Supplementary Fig. S12), thus supporting the functional association of HMGA2 in tumor cells with endothelial cell barrier destruction and formation of a microenvironment conduce to cancer metastasis. Overall, our clinical data suggest that cancer-derived HMGA2 can serve as a blood-based biomarker for the prediction or diagnosis of NPC metastasis.

## Discussion

Increased vascular permeability, a characteristic of tumor vessels, facilitates metastasis, and EBV infection increases the rate of NPC distant metastasis. Concerted efforts in the past two decades have revealed various EBV oncogenic factors, including viral proteins, miRNAs and circRNAs drive EMT and consequently increase the migration, invasion and metastasis of NPC cells. However, the effect of EBV on stromal cells, such as endothelial cells, in the tumor microenvironment remains largely unknown. We revealed that exosomal HMGA2 protein from EBV-positive NPC cells can be delivered into endothelial cells, attenuate endothelial junction integrity, increase EndMT, and consequently promote tumor metastasis. Moreover, serum exosomal HMGA2 can serve as a noninvasive biomarker to predict the metastatic potential of NPC. Gaining insight into the mechanisms of distant metastasis and the specific contribution of tumor-derived exosomes to this process provides opportunities for early diagnosis and therapeutic targeting of NPC.

The EndMT process has been characterized as a transdifferentiation program in which endothelial cells lose their endothelial cell characteristics and gain mesenchymal features. This phenotypic switch is characterized by profound morphological, functional and molecular changes. Endothelial cells lose cellular adhesion and increase permeability [36]. Enhanced vascular permeability can then enhance cancer cell dissemination and growth at distant sites through multiple mechanisms, including (1) enhanced entrapment and hence concentration of tumor cells; (2) enhanced dissemination of tumor cells to distant sites; and (3) leakage of additional exosome cargos and/or plasma proteins into secondary organs and alter cellular physiology toward a prometastatic/tumor-supportive phenotype. EndMT appears to be under the control of several factors such as transforming growth factor-β, basic fibroblast growth factor, Notch, hepatocyte growth factor and platelet-derived growth factor. To date, only one study has used in vitro models to disclose the effect of cancer cell-secreted exosomes on EndMT [37]. Myofibroblastic/mesenchymal cells generated from endothelial cells through EndMT represent a unique source of cancer-associated fibroblasts (CAFs), as initially described in a mouse model [38] First, CAFs can promote the growth, survival and invasiveness of cancer cells as well as angiogenesis. Second, CAFs contribute to the formation of a niche promoting the survival and expansion of extravasated cancer cells. Third, the development of resistance is affected by CAF. Fourth, TGFβ produced by CAF can promote EndMT and increase cell permeability in return [39]. Exosomes derived from the EBV-positive NPC cells have dual effects on endothelial cells, and the coordination of EndMT and endothelial cell permeability promote intravasation, extravasation and colonization of tumor cells.

HMGA2 is a transcriptional factor essential for embryonic development, its expression is decreased during postembryonic development [40–42]. However, HMGA2 levels are often increased during oncogenesis. RNA sequencing analysis of tumor and normal samples has shown that HMGA2 expression is upregulated in most cancers, including head and neck cancer [43, 44]. HMGA2 promotes EMT by inducing the MAPK/ERK, TGFβ/Smad, PI3K/AKT/mTOR/NF-kB, and RKIP pathways, as well as regulating miRNA expression [45]. High HMGA2 expression also promotes cell proliferation and invasion in nasopharyngeal carcinoma [43, 44]. However, how HMGA2 affects the tumor microenvironment is unclear. We found that exosomal HMGA2 derived from EBV-positive NPC cells can be internalized by endothelial cells with low HMGA2 expression and subsequently promote endothelial cell permeability and EndMT, which means that HMGA2 has function in enhancing tumor cell invasion (of tumor cells themselves) and weakening endothelial barriers (the tumor microenvironment). Our findings broaden the understanding of HMGA2 in cancer metastasis.

Recent studies have revealed the emerging role of exosomal biomarkers in cancer diagnosis and prognosis assessment. Exosomal CD44v6/C1QBP from pancreatic ductal adenocarcinoma cells contributes to premetastatic niche formation in the liver and may be used for the diagnosis of pancreatic ductal adenocarcinoma liver metastasis [46]. Exosomal integrins have been verified to be potential markers for predicting organ-specific metastasis [47]. Of note, it has been reported that M2 macrophage-derived exosomal α_M_ β_2_ integrin facilitates HCC metastasis [48]. The clinical relevance of HMGA2 in NPC tissue with distant metastasis is underscored by its significant negative correlation with TJ protein expression and its positive association with mesenchymal biomarker expression. Moreover, a high level of HMGA2 in serum exosomes was associated with metastatic progression, suggesting that serum exosomal HMGA2 may be a reliable biomarker of metastatic risk in NPC.

HMGA2 has been shown to be involved in cancer stemness in different types of cancers [49, 50]. Recent studies have suggested that EBV-encoded proteins could induce stem cell-like properties in NPC [51, 52]. This may explain why HMGA2 was overexpressed in EBV-positive NPC cells compared with EBV-negative NPC cells and why HMGA2 was abundant in exosomes derived from EBV-positive NPC cells compared with exosomes derived from EBV-negative NPC cells. Although the function of HMGA2 in EMT has been reported, the function of HMGA2 in EndMT in endothelial cells is unknown. One article reported a potential correlation between HMGA2 and EndMT in acute aortic dissection [53], but its function in EndMT was not validated by experiments. In this study, we demonstrated that HMGA2 functionally regulates EndMT.

Overall, our findings identify a role for HMGA2 in NPC distant metastasis via altering tumor microenvironment, suggesting that it is a promising prognostic biomarker and therapeutic target for metastasis. Our future studies will focus on circulating tumor-derived exosomal HMGA2 as a plasma-based biomarker for noninvasive screening of patients for NPC metastases, along with testing targeted therapies aimed at blocking HMGA2 to halt metastasis.

## Supplementary information

Supplementary Informations

Supplementary Figure 1

Supplementary Figure 2

Supplementary Figure 3

Supplementary Figure 4

Supplementary Figure 5

Supplementary Figure 6

Supplementary Figure 7

Supplementary Figure 8

Supplementary Figure 9

Supplementary Figure 10

Supplementary Figure 11

Supplementary Figure 12
