# Supplementary material for: Exosomal HMGA2 protein from EBV-positive NPC cells destroys vascular endothelial barriers and induces endothelial-to-mesenchymal transition to promote metastasis
Source: Cancer Gene Ther. 2022 Apr 6;29(10):1439–51. doi: 10.1038/s41417-022-00453-6 (PMC9576596; doi:10.1038/s41417-022-00453-6)
Supplement: Supplementary file 1 — Supplementary Informations [file 41417_2022_453_MOESM1_ESM.docx]

**Supplementary figure legends**

**Supplementary 1. Characteristic of NPC cells derived exosomes**

A. Transmission electron microscopy of exosomes isolated from NPC cells.

B. The concentration and size of exosome were determined by nanoparticle tracking analysis (NTA).

C. Western blotting demonstrated the presence of the common exosome markers: Syntenin-1 and CD63, as well as Calnexin in the endoplasmic reticulum and VDAC1 in mitochondrion. A total of 3 µg of protein was loaded in each lane.

**Supplementary Figure 2. Inhibition of exosome uptake with EIPA attenuated the reductions in TJ protein expression and EndMT in endothelial cells.**

A. Representative images (high panel) and histogram of the quantification (low panel) of the endothelial cellular morphology alteration after the cells were treated as indicated for 48 hr. ***p < 0.001.

B. The permeability of treated HUVEC monolayers was measured by the appearance of rhodamine-dextran, which was added to the top well at the beginning of the experiment, in the bottom well during a 2 hr time course. The absorbance at 590 nm at each time point is indicated. ***p < 0.001.

C. HUVECs treated with PBS, EIPA, NPC43-Exo, EIPA and NPC43-Exo were analyzed by Western blotting for the TJ proteins.

D. HUVECs treated by PBS, EIPA, NPC43-Exo, EIPA and NPC43-Exo were analyzed by Western blotting for CD31, α-SMA, Vimentin and S100A4.

**Supplementary Figure 3. Exosomes can be internalized by endothelial cells in vivo.**

The uptake of HK1-Exo and NPC43-Exo by endothelial cells was measured by colocalization of PKH67-labeled exosomes (green) and the endothelial cell marker CD31 (red). The arrows show colocalization (scale bar = 20 μm).

**Supplementary Figure 4.** **Changes in endothelial cellular TJ protein levels in vivo.**

Nude mice were treated exosomes derived from the indicated cells a total of four times at an interval of two days. Collected lung and liver tissues were subjected to double-label immunofluorescence for ZO-1 (green), occludin (green), and claudin-5 (green) and CD31 (red). Structures positive for CD31 are indicated by arrowheads. The scale bar represents 100 μm.

**Supplementary Figure 5. Analysis of proteins that is higher in NPC43 cells derived exosomes compared with HK1 cells derived exosomes**

A. Venn diagram of proteins enriched in exosomes derived from NPC43 cells (> 2 times) compared to exosomes derived from HK1 cells identified by label-free proteomics analysis (the mass spectrum data were repeated three times).

B. Classification of 181 proteins enriched in NPC43 cell derived exosomes represented as a bar chart based on protein class.

C. List of 19 proteins involved in EMT, the actin cytoskeleton, focal adhesion and TJs with significantly higher expression in NPC43 cell-derived exosomes than in HK1 cell-derived exosomes out of the 181 enriched proteins.

**Supplementary Figure 6. The expression and function of candidate proteins**

A. Western blot analysis showed the content of DDX6, CEMIP, CD82 and MTA2 in exosomes derived from EBV-negative NPC cells and exosomes derived from EBV-positive NPC cells.

B. Overexpression of five candidate proteins in HUVECs was detected by Western blotting.

C. Representative cellular morphology alterations (high panel) and spindle-shape ratios (low panel) of endothelial cells overexpressed with overexpressing HMGA2, DDX6, CEMIP, CD82 and MTA2 respectively. The spindle-shaped cells are marked with a black line. ***p < 0.001.

D. The permeability of the indicated HUVEC monolayers grown on 0.4 μm filters was measured by the appearance of rhodamine-dextran. ***p < 0.001.

**Supplementary Figure 7. HMGA2 overexpression in HK1 cells and HMGA2 knockdown in NPC43 cells have no significant effect on exosome production or protein content.**

A. After overexpression and knockdown of HMGA2 in HK1 and NPC43 cells, exosomes were investigated by transmission electron microscopy.

B, C and D. Size distribution and protein content analysis of exosomes derived from HK1 cells with HMGA2 overexpression and NPC43 cells with HMGA2 knockdown. Exosome size and number were evaluated by NTA. The protein content per exosome ([particle]/[protein]) was assessed on the basis of the protein concentration. The presence of common exosome markers was demonstrated by Western blotting. TEM images (A) are representative of three independent biological replicates. Scale bars, 200 nm. Error bars depict the mean ± SD. P values were calculated by ANOVA.

**Supplementary Figure 8. The expression of HMGA2 in EBV-negative NPC cells, EBV-positive NPC cells and HUVECs treated with the indicated cell-derived exosomes.**

A. Western blot analysis showed the expression of HMGA2 in EBV-negative NPC cells (HK1, HONE1, SUNE1, HNE1, CNE2, TW03 and 5-8F cells), EBV-positive NPC (NPC43, C666-1 and C17 cells) and HUVECs.

B. HUVECs were treated with HK1-Exo, CNE2-Exo, NPC43-Exo, C666-1-Exo and C17-Exo for 6 hr and 18 hr, and then q-PCR was used to detect HMGA2 mRNA in the HUVECs.

**Supplementary Figure 9. Analysis of signaling pathway and genes affected by HMGA2**

A. Top, pathways affected by NPC43 cells derived exosomes in HUVECs compared with HK1 cells derived exosomes and PBS. Bottom, a heatmap

of differentially expressed genes involved in selected pathways.

B. Top, pathways affected by HMGA2 in HUVECs with HMGA2 overexpression compared with the control. Bottom, a heatmap of differentially expressed genes involved in selected pathways.

**Supplementary Figure 10.** **Changes of endothelial cellular TJ protein levels in the liver and lung.**

Nude mice were treated with exosomes derived from the indicated cells every other day for 2 weeks. Collected lung and liver tissues were subjected to double-label immunofluorescence for ZO-1 (green), occludin (green), claudin-5 (green) and CD31 (red). Structures positive for CD31 are indicated by arrowheads. The scale bar represents 100 μm.

**Supplementary Figure 11. The change in endothelial cellular TJ protein levels in the lung.**

Tumor lumps derived from subcutaneous 5-8F control or 5-8F HMGA2 OE tumors in mice were transferred into the livers of nude mice. Collected lung tissues were subjected to double-label immunofluorescence for ZO-1 (green), occludin (green) and claudin-5 (green) and CD31 (red). Structures positive for CD31 are indicated by arrowheads. The scale bar represents 100 μm.

**Supplementary Figure 12. Immunohistochemistry shows the relationship between HMGA2 in NPC tumor cells and TJ proteins, EndMT markers of endothelial cells**

Serial sections of non-NPC tumor tissues, NPC tissues with low HMGA2 expression and NPC tissues with high HMGA2 expression were stained with H&E and antibodies targeting Snail, CD31, TJ proteins, α-SMA, Vimentin and S100A4. Scale bars: 50 μm. Spearman correlation analysis between HMGA2 in tumor cells and above protein expression in endothelial cells. Pearson’s correlation coefficient (r) and P-value are shown. P-value is from Spearman’s test.
