# Supplementary figures and images for: Exosomal HMGA2 protein from EBV-positive NPC cells destroys vascular endothelial barriers and induces endothelial-to-mesenchymal transition to promote metastasis
Source: Cancer Gene Ther. 2022 Apr 6;29(10):1439–51. doi: 10.1038/s41417-022-00453-6 (PMC9576596; doi:10.1038/s41417-022-00453-6)

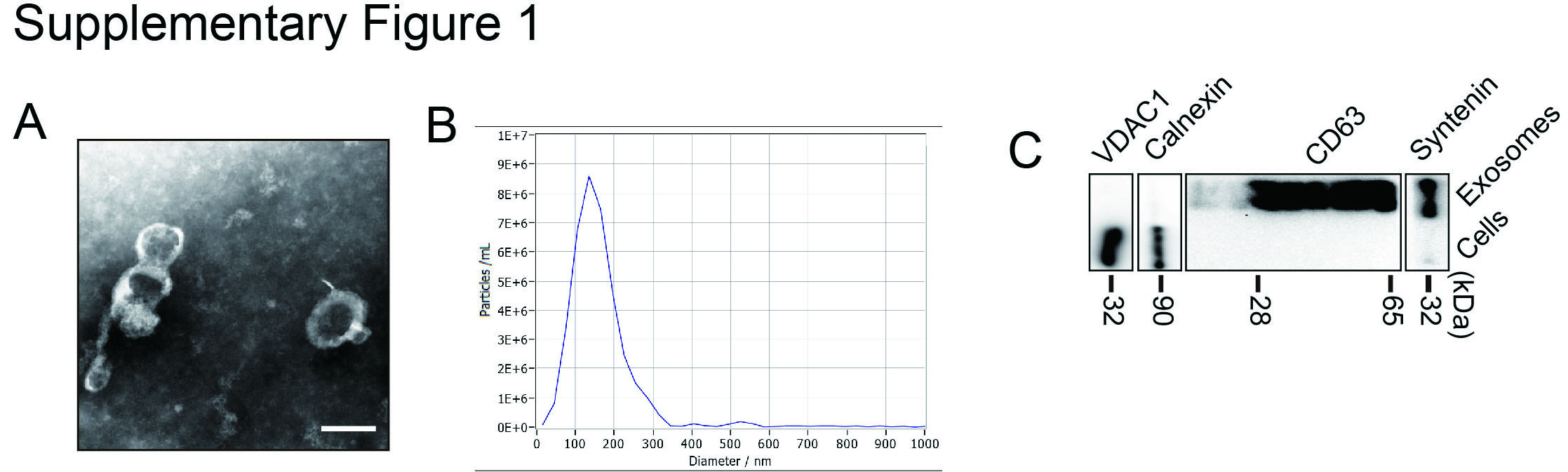

Supplement: Supplementary file 2 — Supplementary Figure 1 [file 41417_2022_453_MOESM2_ESM.jpg]

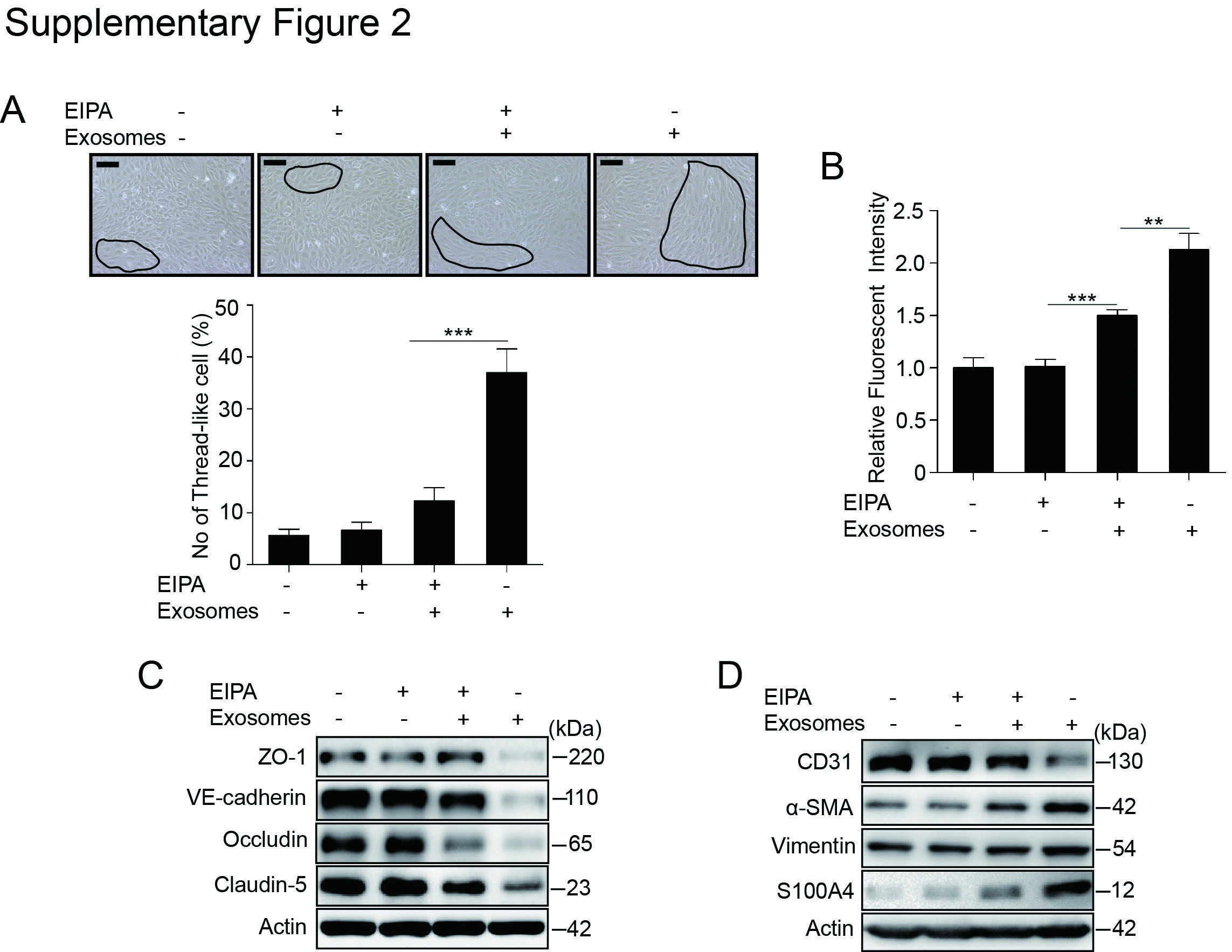

Supplement: Supplementary file 3 — Supplementary Figure 2 [file 41417_2022_453_MOESM3_ESM.jpg]

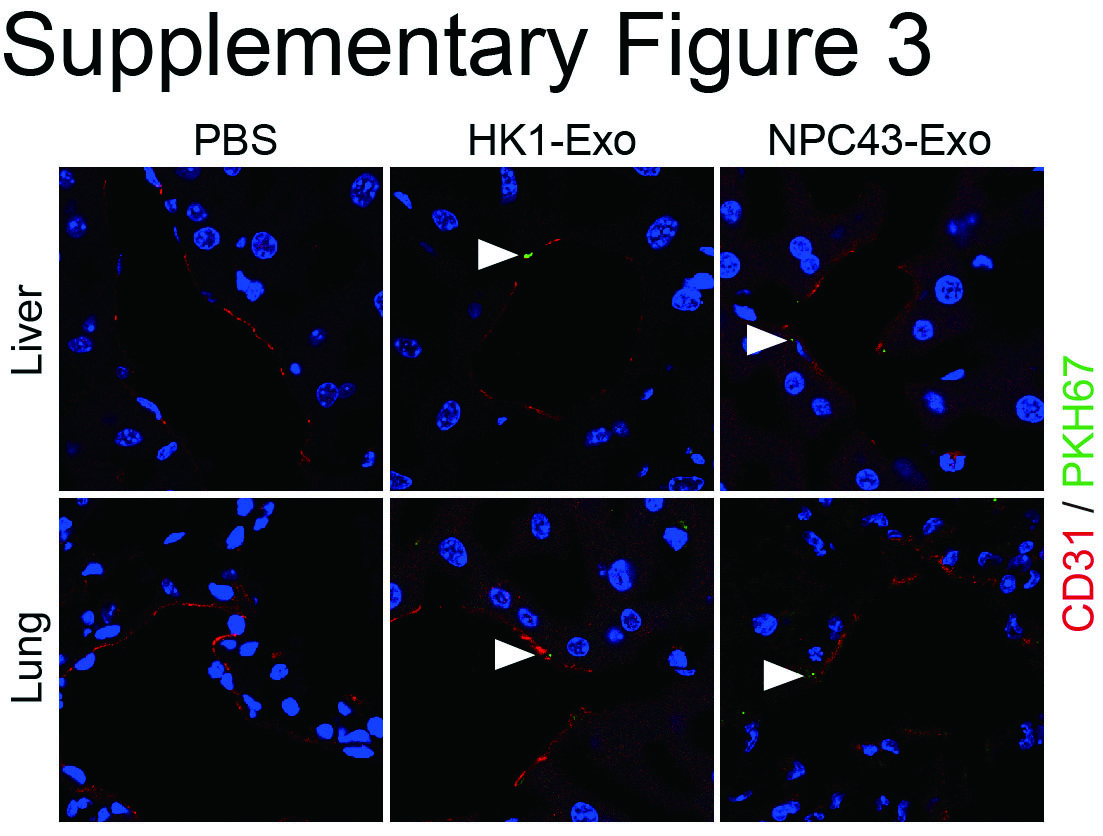

Supplement: Supplementary file 4 — Supplementary Figure 3 [file 41417_2022_453_MOESM4_ESM.jpg]

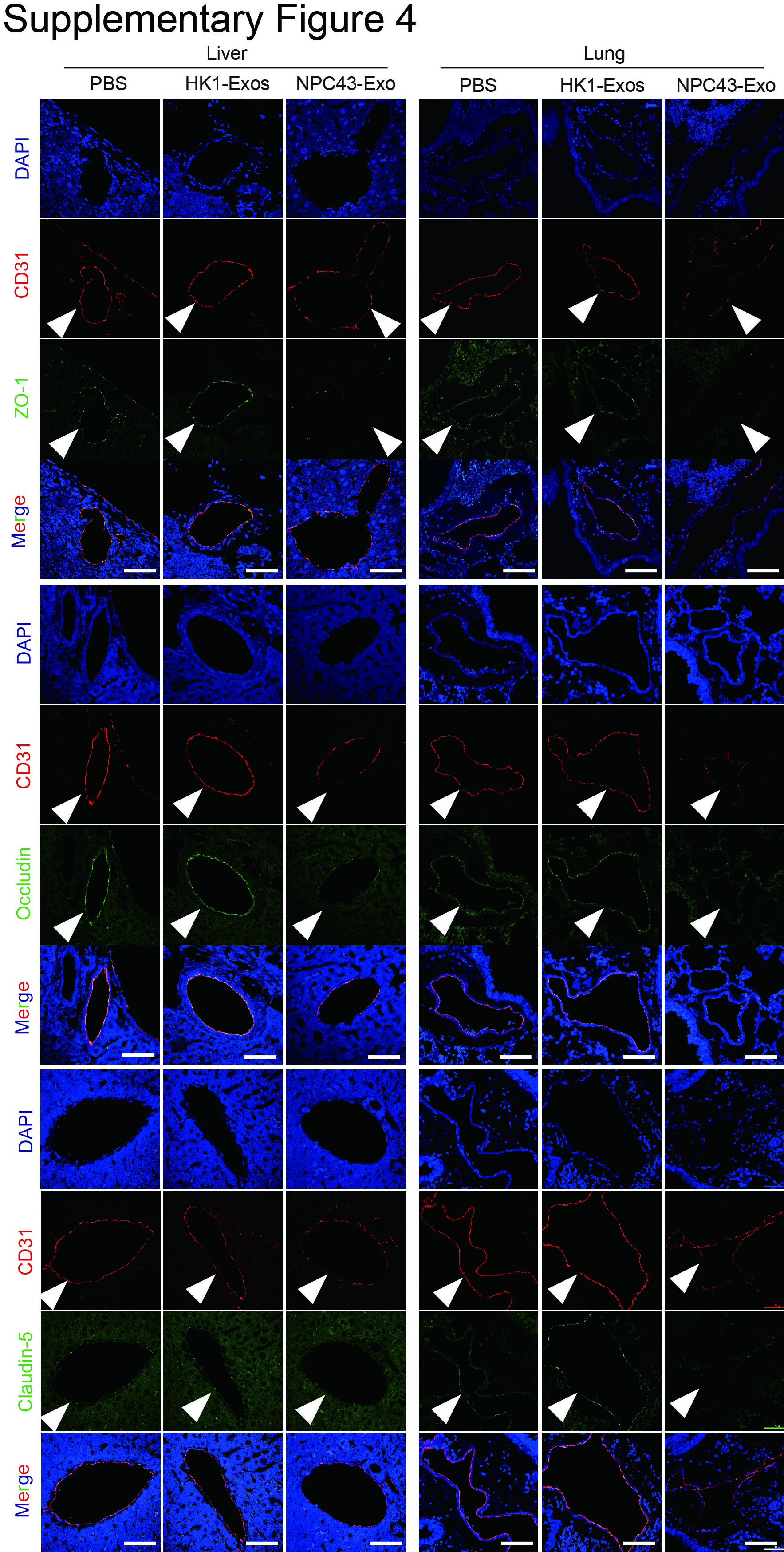

Supplement: Supplementary file 5 — Supplementary Figure 4 [file 41417_2022_453_MOESM5_ESM.jpg]

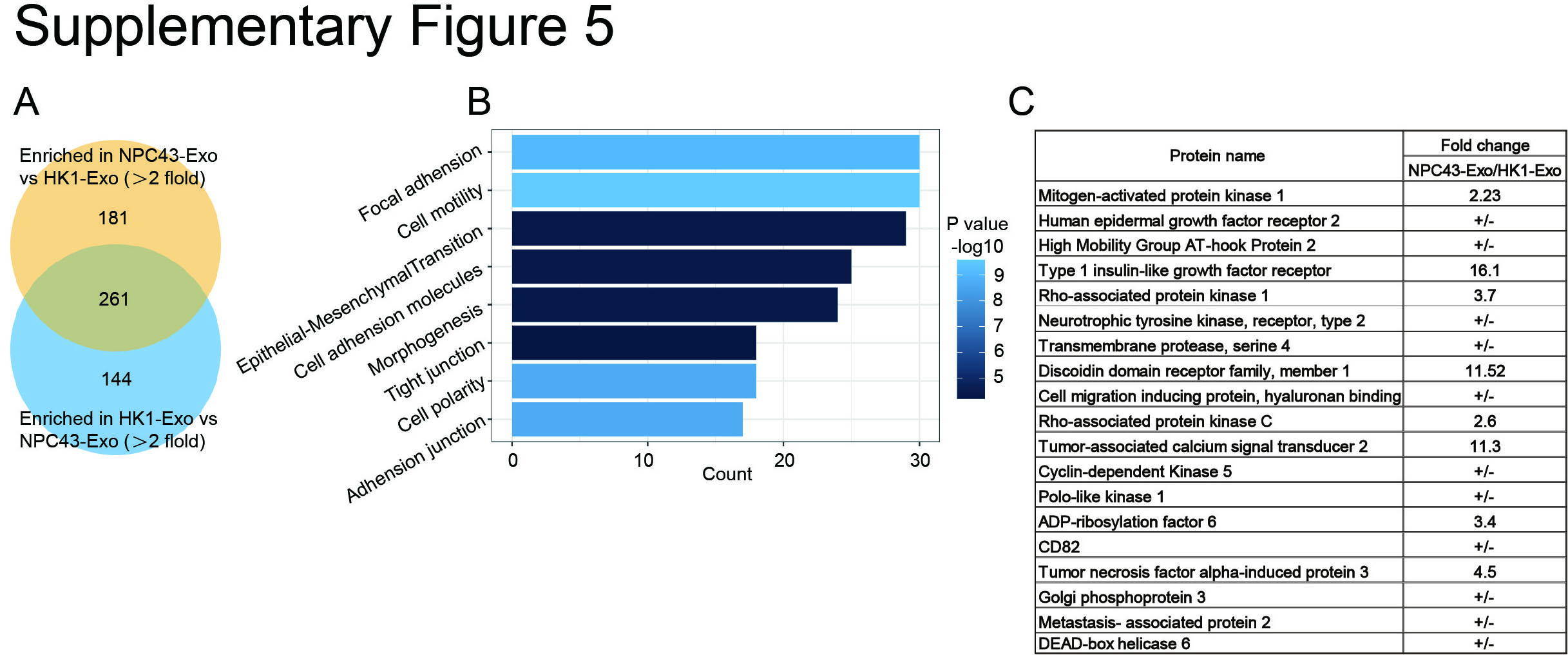

Supplement: Supplementary file 6 — Supplementary Figure 5 [file 41417_2022_453_MOESM6_ESM.jpg]

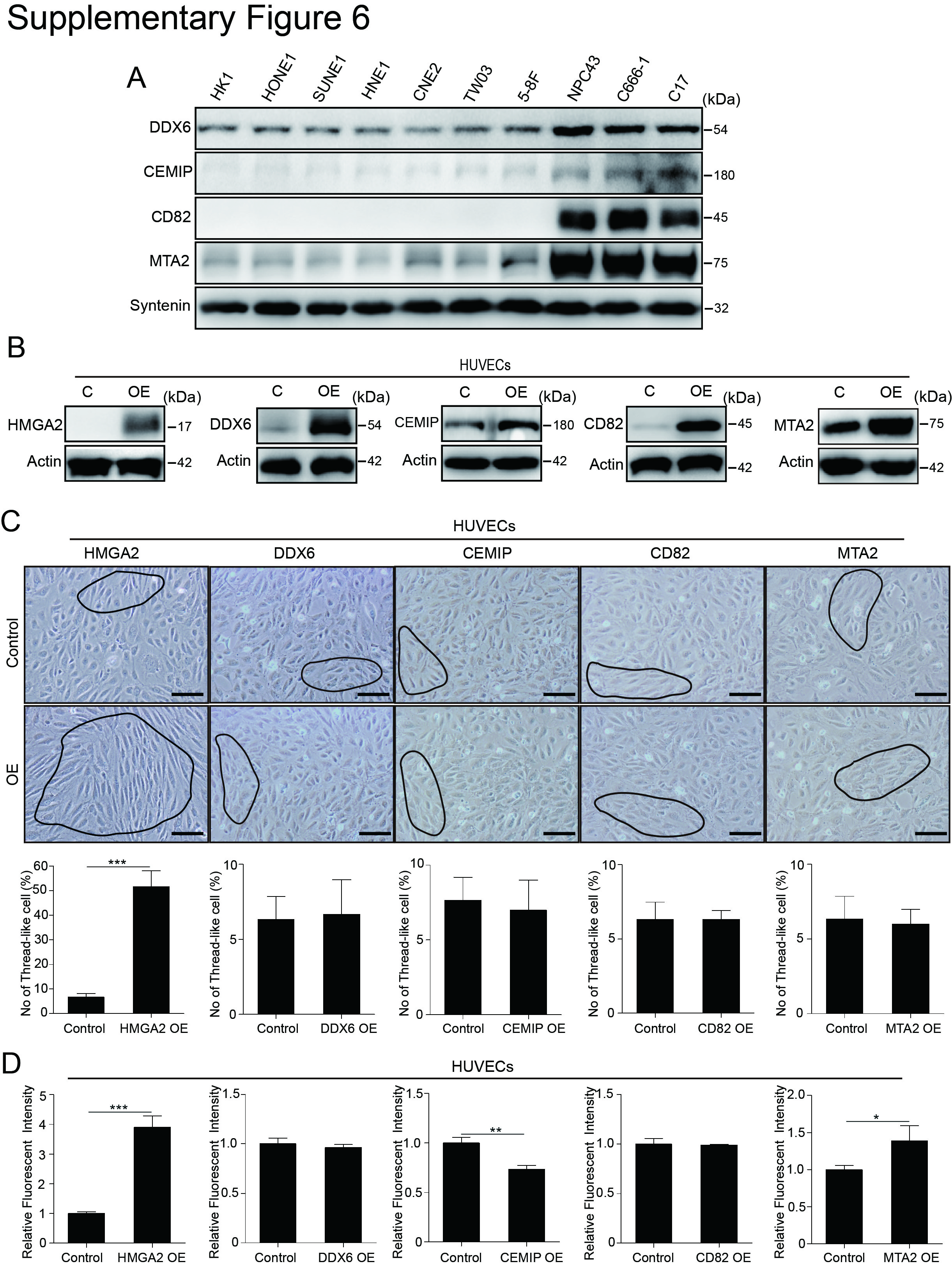

Supplement: Supplementary file 7 — Supplementary Figure 6 [file 41417_2022_453_MOESM7_ESM.jpg]

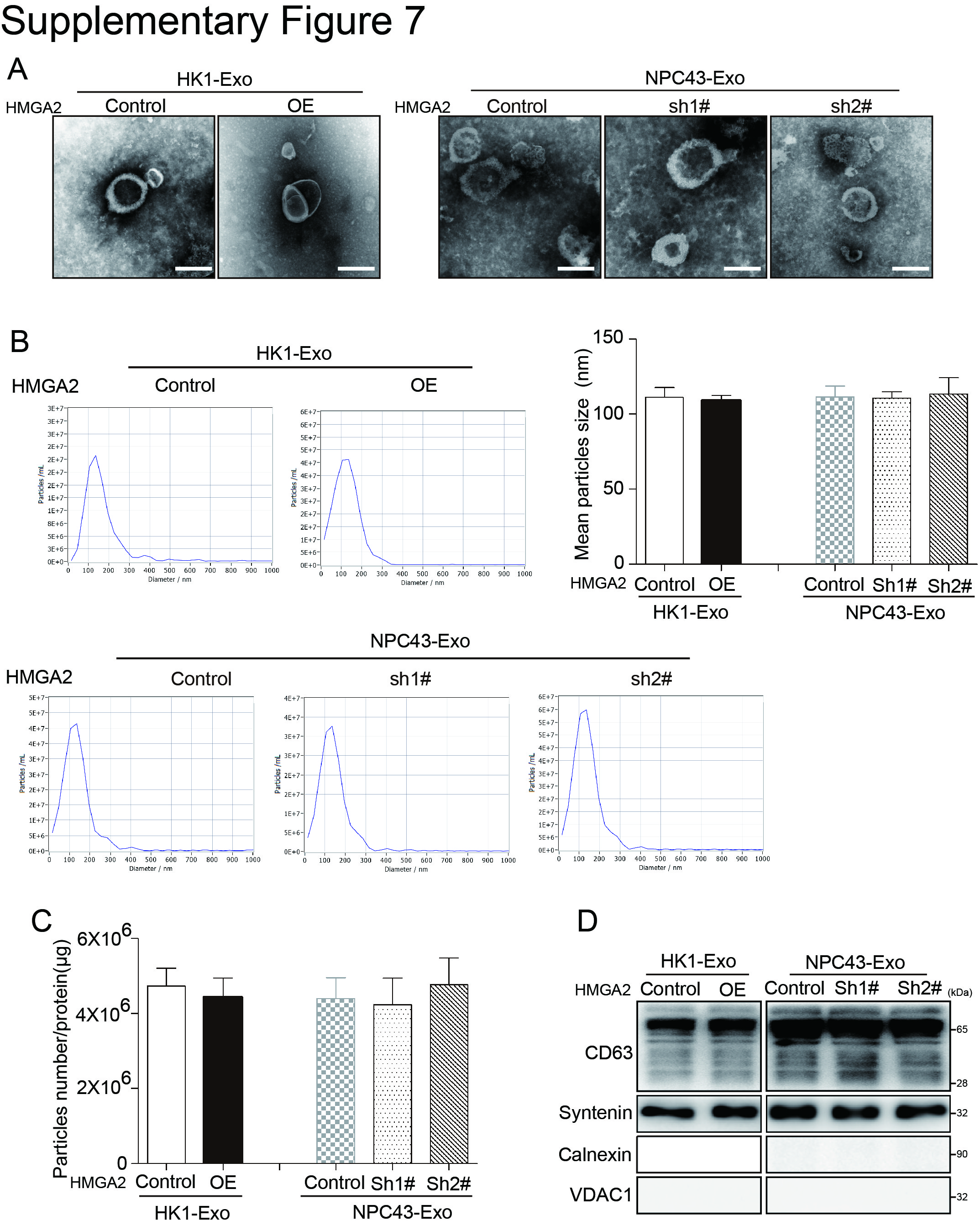

Supplement: Supplementary file 8 — Supplementary Figure 7 [file 41417_2022_453_MOESM8_ESM.jpg]

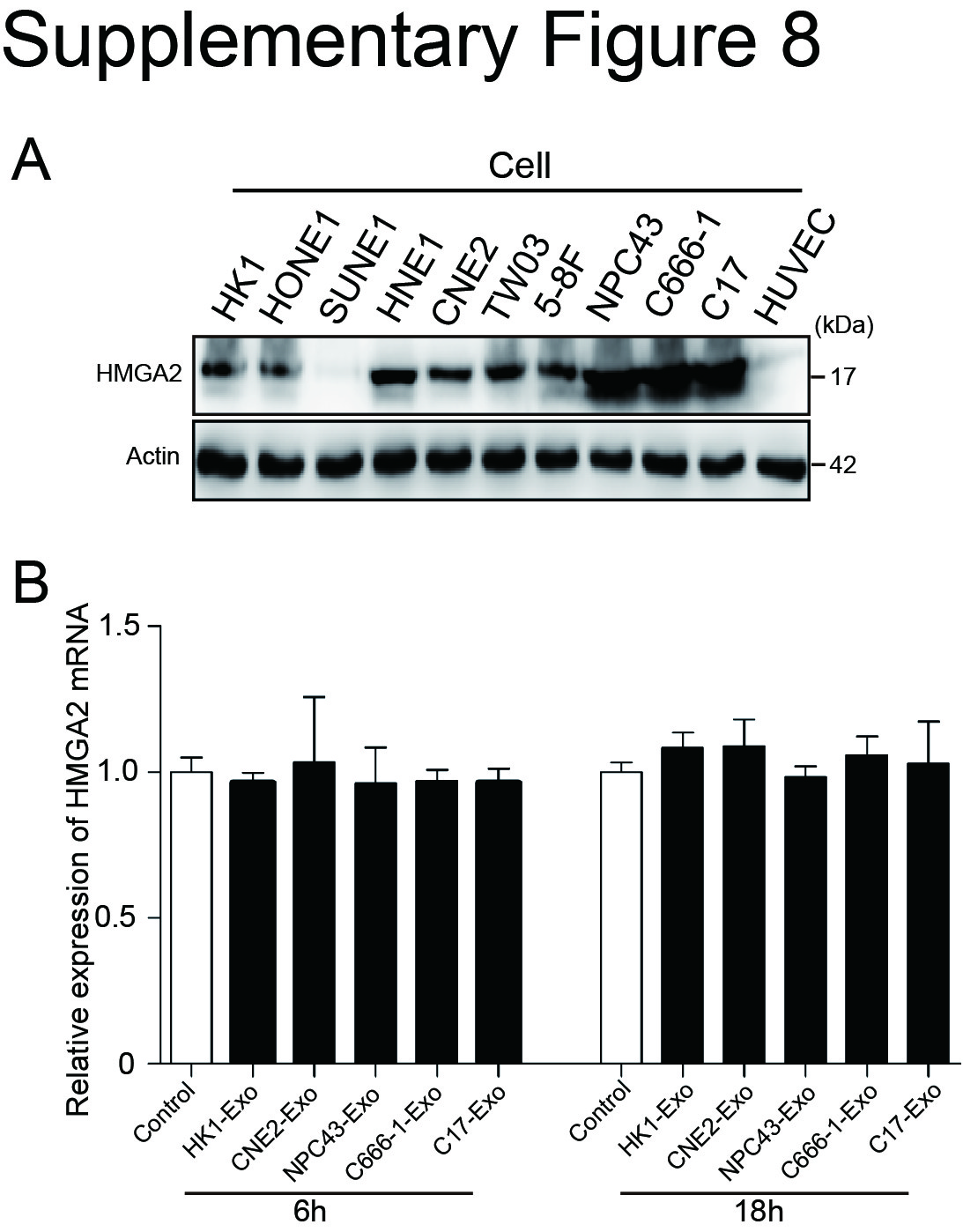

Supplement: Supplementary file 9 — Supplementary Figure 8 [file 41417_2022_453_MOESM9_ESM.jpg]

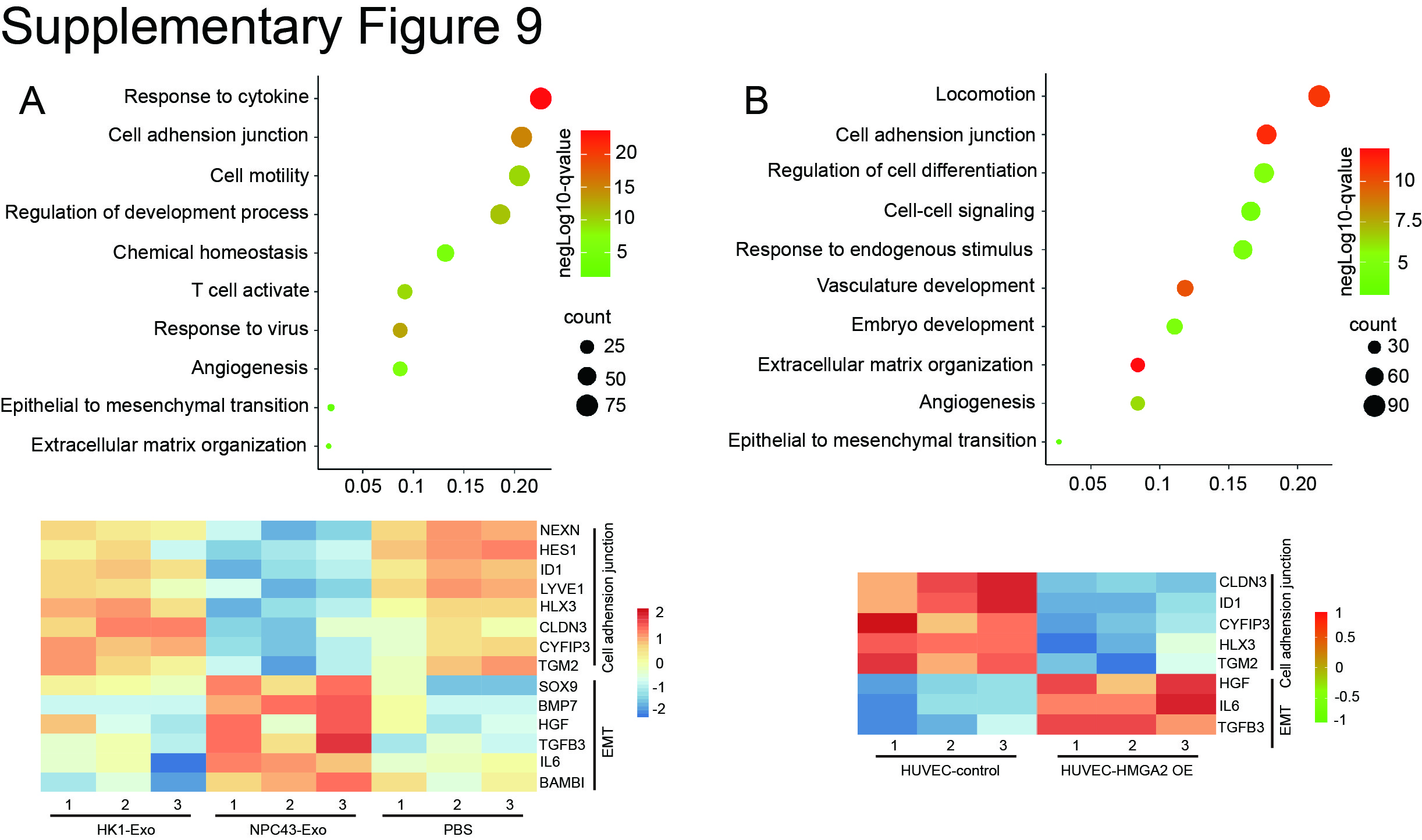

Supplement: Supplementary file 10 — Supplementary Figure 9 [file 41417_2022_453_MOESM10_ESM.jpg]

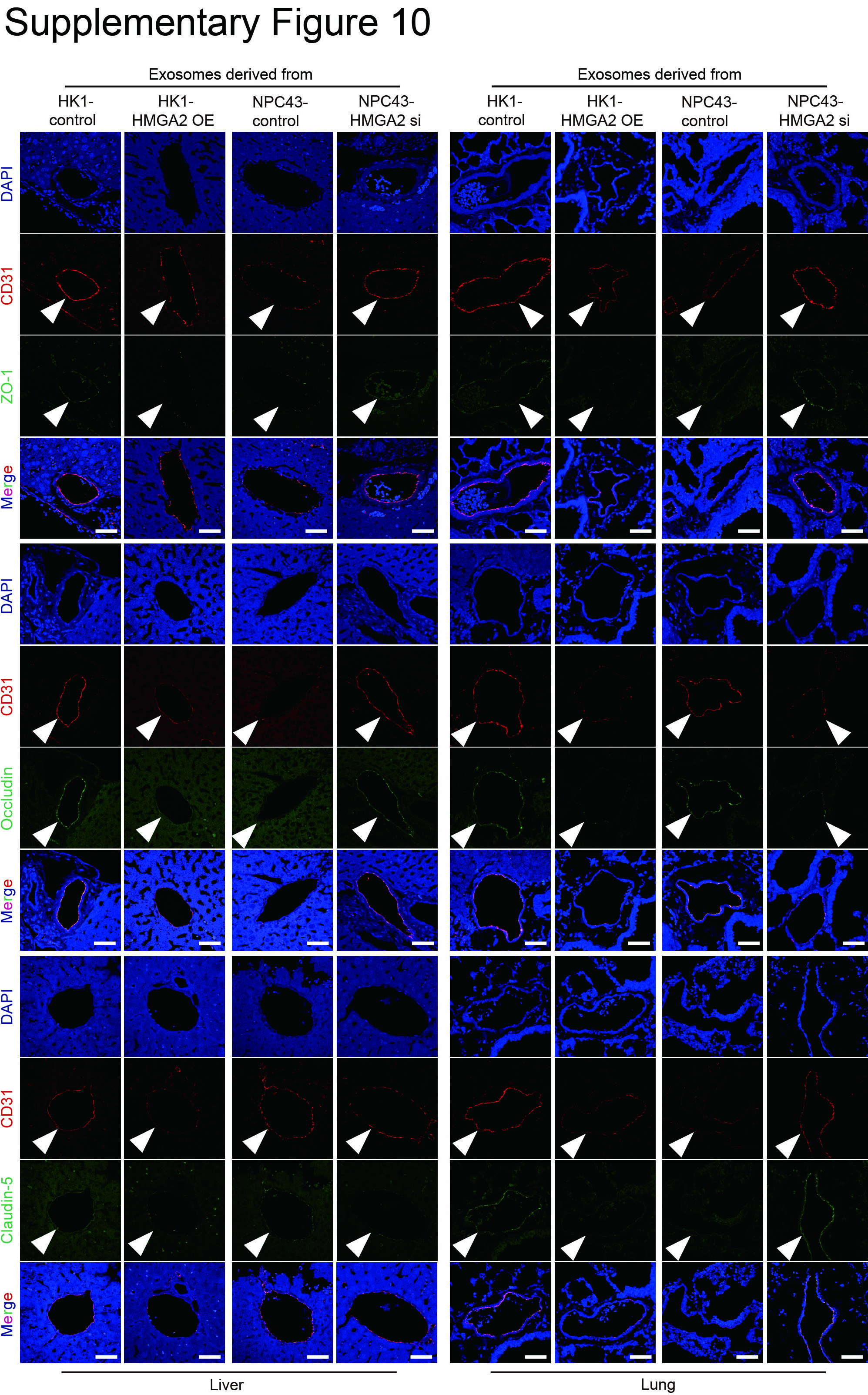

Supplement: Supplementary file 11 — Supplementary Figure 10 [file 41417_2022_453_MOESM11_ESM.jpg]

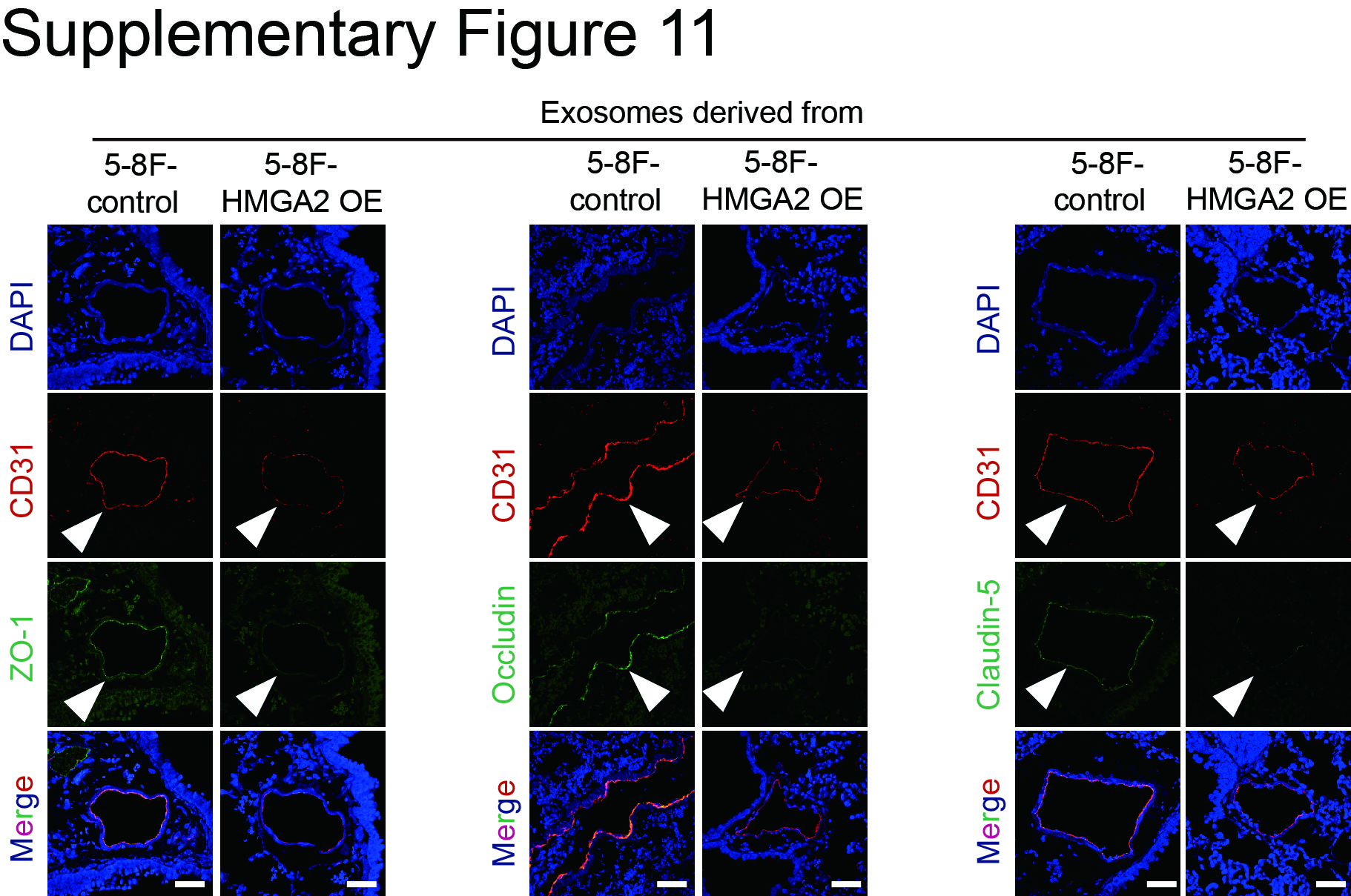

Supplement: Supplementary file 12 — Supplementary Figure 11 [file 41417_2022_453_MOESM12_ESM.jpg]

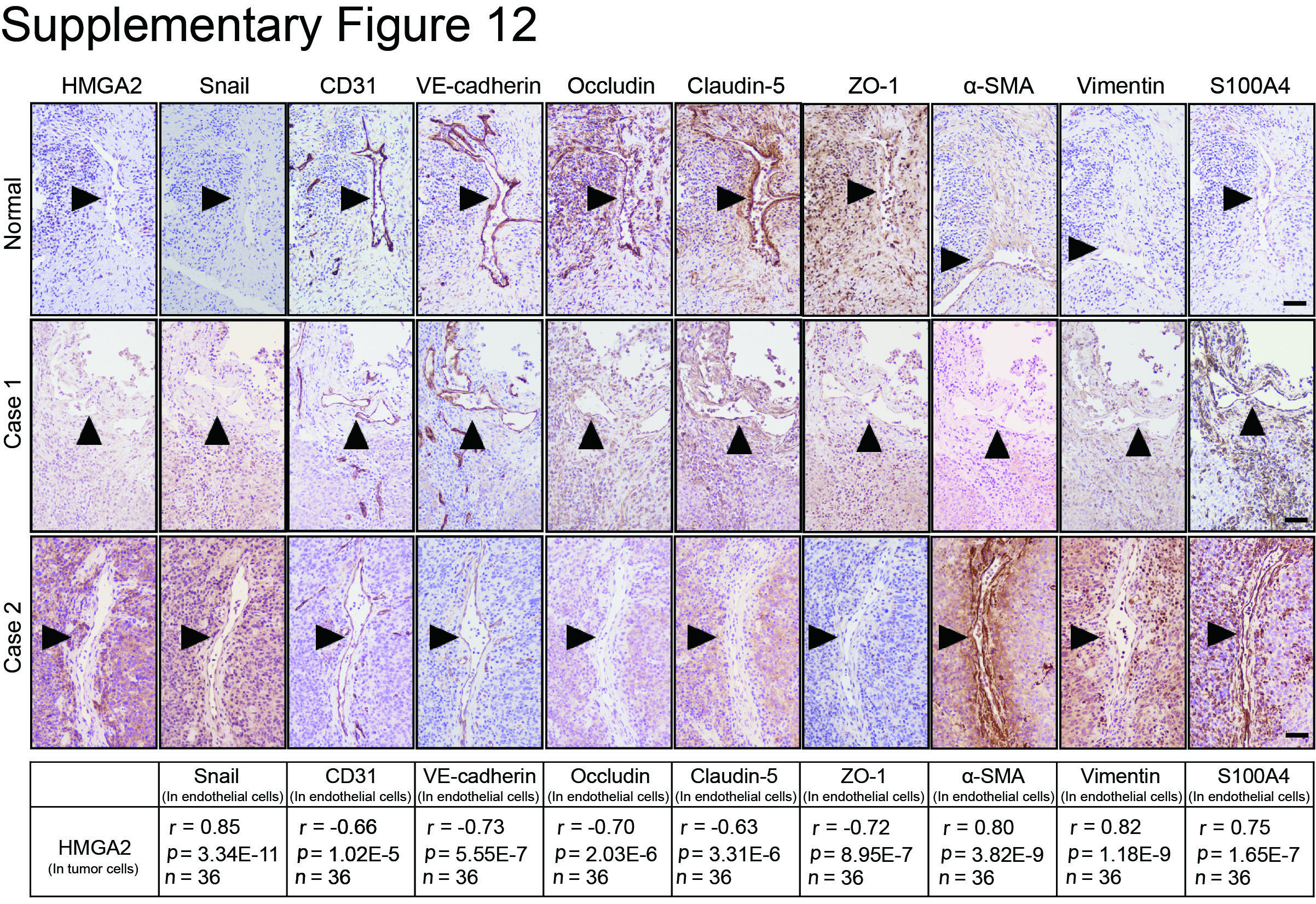

Supplement: Supplementary file 13 — Supplementary Figure 12 [file 41417_2022_453_MOESM13_ESM.jpg]
